# Adaptive Manufacturing for Healthcare During the COVID-19 Emergency and Beyond

**DOI:** 10.3389/fmedt.2021.702526

**Published:** 2021-08-02

**Authors:** Antoine Vallatos, James M. Maguire, Nikolas Pilavakis, Gabrielis Cerniauskas, Alexander Sturtivant, Alexander J. Speakman, Steve Gourlay, Scott Inglis, Graham McCall, Andrew Davie, Mike Boyd, Adriana A. S. Tavares, Connor Doherty, Sharen Roberts, Paul Aitken, Mark Mason, Scott Cummings, Andrew Mullen, Gordon Paterson, Matthew Proudfoot, Sean Brady, Steven Kesterton, Fraser Queen, Steve Fletcher, Andrew Sherlock, Katherine E. Dunn

**Affiliations:** ^1^Centre for Clinical Brain Sciences, Chancellor's Building, University of Edinburgh, Edinburgh, United Kingdom; ^2^School of Engineering, University of Edinburgh, Edinburgh, United Kingdom; ^3^School of Informatics, University of Edinburgh, Edinburgh, United Kingdom; ^4^Department of Medical Physics, NHS Lothian, Royal Infirmary of Edinburgh, Edinburgh, United Kingdom; ^5^AESSiS - Advanced Engineering Solutions, London, United Kingdom; ^6^uCreate Studio, Main Library, University of Edinburgh, George Square, Edinburgh, United Kingdom; ^7^British Heart Foundation/University of Edinburgh Centre for Cardiovascular Science and Edinburgh Imaging, Queen's Medical Research Institute, University of Edinburgh, Edinburgh, United Kingdom; ^8^Lomond Process Engineering, Glasgow, United Kingdom; ^9^Gemini Digital Technologies, Northwich, United Kingdom; ^10^Shapespace, Edinburgh, United Kingdom

**Keywords:** COVID-19, reverse engineering, CAD, 3D printing, product lifecycle management, additive manufacture

## Abstract

During the COVID-19 pandemic, global health services have faced unprecedented demands. Many key workers in health and social care have experienced crippling shortages of personal protective equipment, and clinical engineers in hospitals have been severely stretched due to insufficient supplies of medical devices and equipment. Many engineers who normally work in other sectors have been redeployed to address the crisis, and they have rapidly improvised solutions to some of the challenges that emerged, using a combination of low-tech and cutting-edge methods. Much publicity has been given to efforts to design new ventilator systems and the production of 3D-printed face shields, but many other devices and systems have been developed or explored. This paper presents a description of efforts to reverse engineer or redesign critical parts, specifically a manifold for an anaesthesia station, a leak port, plasticware for COVID-19 testing, and a syringe pump lock box. The insights obtained from these projects were used to develop a product lifecycle management system based on Aras Innovator, which could with further work be deployed to facilitate future rapid response manufacturing of bespoke hardware for healthcare. The lessons learned could inform plans to exploit distributed manufacturing to secure back-up supply chains for future emergency situations. If applied generally, the concept of distributed manufacturing could give rise to “21st century cottage industries” or “nanofactories,” where high-tech goods are produced locally in small batches.

## Introduction

The COVID-19 pandemic caused critical global supply shortages of equipment and material, ranging from personal protective equipment (masks, face shields, gloves, aprons, etc) to hardware such as ventilators (1, 2). The field of medical technology had been thriving before the pandemic (3) but the pandemic created a pressing need for extremely rapid innovation and improvisation, with limited resources, in a high-stakes situation (4).

One hospital in Japan developed a way to turn a refuse bag into a long-sleeved plastic gown, which could be worn for various procedures including endoscopies (5). As will be described in more detail below, 3D printing was used by people around the globe to make a range of items, from face shields to swabs for testing (6). A particularly notable example from the early stages of the first wave of the pandemic in Europe was the development by Italian company Isinnova of 3D-printed components that enabled a snorkelling mask to be connected to a ventilator and used in intensive care units (7). Also in connexion with respiratory support, a large number of individuals and groups from various organisations worked on new emergency ventilators or continuous positive airway pressure (CPAP) systems (8–10). In some cases, novel methods were used to speed up the development process, such as parallel testing of alternative designs (8).

These and other projects emerged as a result of the near-collapse of conventional supply chains and inability of the normal manufacturers to meet demand. Many of the efforts were driven by altruism and a desire to help with the ongoing crisis. Some of the participants in these projects were hobbyists with little or no experience in the medical technology field. Ideas and designs were often shared freely via the Internet, giving rise to “open source hardware” (11, 12), which was manufactured in many locations (“distributed manufacturing”). This distributed manufacturing was built on the enthusiasm of a broad community of participants who were able to respond more rapidly than formal organisations. As an aside, it is important to note distributed manufacturing often involves production of items very close to the point of use, in contrast to more conventional factory-based production. It is therefore possible to speculate that the development of “21st century cottage industries” or “nanofactories” based on distributed manufacturing (for various commodities, not just medical devices and not necessarily open-source hardware) might help to reduce the carbon footprint of the manufacturing sector, by reducing the distance over which goods must be moved. However, this would need to be evaluated on a case-by-case basis with a full life cycle assessment, taking into account factors such as material transport requirements, material wastage, energy use during manufacture, product lifetime and end-of-life disposal. It is conceivable that additively manufactured components could be more environmentally damaging than alternatives produced in the traditional manner.

In some COVID-19 emergency projects, items were designed from scratch (forward engineering), while in other cases hardware was reverse-engineered from existing equipment (13). Sometimes a mixture of the two approaches was used. Forward engineering refers to the traditional design-build-test-learn process, where the designer starts with a conceptual design and finishes with a physical product. In contrast, for reverse engineering the process begins with a physical product, and works back toward the detailed design stage. In reverse engineering, the features and surfaces are captured using a variety of methods including scanning, to generate an initial digital representation (14, 15). This initial representation must then be converted into a usable 3D “as manufactured” model format by point processing and remodelling, using a range of algorithms. A design model can be generated to capture design intent. Finally a CAD file can be used for manufacture of a duplicate or evolved version of the original product. Reverse engineering can be used for a range of applications, such as the replacement of machine parts that are obsolete and unobtainable, analysing competitors' products, quality control, reconstructing designs when the original information has been lost, and so on (14, 15). Although a number of legal issues may arise with reverse engineering, the techniques are of interest in various sectors, including the automotive industry (16) and healthcare, as will be seen. In some cases, regulators might be able to approve a reverse-engineered product more rapidly than an unproven *de novo* design, on the basis of equivalence between the reverse-engineered product and the pre-approved original.

The physical-to-digital transition involves capturing the form and 2D/3D geometry of the target object or assembly of objects. This can be accomplished a number of non-contact 3D scanning solutions including laser line (17, 18) scanning, structured light scanning (19) or CT technologies.

3D scanning captures the surfaces of a component by projecting light on to the object in the form of a line or pattern which is captured by the optical sensor, and interpreted using software and algorithms both embedded within the sensor and in an external application. 3D point clouds are produced and stitched together to create a digital representation of the physical object. Highly reflective or transparent surfaces can be covered with a non-invasive chalk type spray solution to enable sufficient light to be reflected without changing the shape of the object. For some applications optical scanning is insufficient and it is necessary to use CT (computed tomography) (20), an X-ray based technique that is also used in medical imaging. This is particularly true for highly reflective or transparent parts, if the chalk spray technique should fail to enable sufficient resolution from optical scanning. One example of the use of CT scanning in the reverse-engineering process is provided by the UCL Ventura device, for which the geometry of pre-existing CPAP devices was verified using CT scans (10).

If a replica part is required for a given application, a suitable production technique must be identified. Manufacturing techniques for consumer goods and medical devices include injection moulding, vacuum forming, CNC machining, casting and many others. In the context of the present work, the most interesting technique for the replication of parts is 3D printing, a type of additive manufacturing which builds objects layer-by-layer (21). In forward engineering, 3D printing is often limited to rapid prototyping prior to scaled-up manufacturing of the finished product using a conventional manufacturing process. As will be presented in this paper, however, when combined with reverse engineering, 3D printing can act as a highly adaptable alternative to conventional manufacturing processes in emergency situations. One 3D printing method is stereolithography (SLA), which involves use of a laser beam to solidify the photoreactive printing resin in layers (22). An alternative 3D printing technique uses a heated nozzle to extrude and deposit molten polymer layer-by-layer onto a print bed; this is known as fused deposition modelling (FDM).

Prior to the pandemic, 3D printing was already established in some sectors of the medical community for applications in surgery and teaching, and the use of 3D printed parts in surgery can sometimes reduce the time spent in the operating room by as much as an hour (23). As noted above, 3D printing was applied much more broadly during the pandemic (13), from face shields (24) to nasal swabs for testing (25, 26) and parts for systems that provide emergency breathing support (6). This was necessary because the unprecedented demand had exhausted the conventional supply networks. One study from June 2020 indicated the potential scale of the problem, estimating that, for some types of hardware, there would be a need for billions of individual 3D printed items (27).

However, the demand for 3D printed face shields fell off after a few weeks (based on personal experience of some of the authors of this paper) as injection moulding was brought online. 3D printing is better suited for low volume production and injection moulding offered advantages in terms of cost, time, labour, and quality control. Effectively, 3D printing was deployed to bridge the gap between urgent demand and scaled-up production (27). Normally, technology paradigm shifts tend to occur years apart, but in this specific case the transition occurred over a few weeks, accompanied by the partial recovery of the normal supply routes. The peak in demand for 3D printed hardware and subsequent fall-off was also seen by a group in Barcelona, who established an online catalogue of 3D printed items that could be ordered by local hospitals (28). Ultimately they distributed over 19,000 items, validated with an appropriate ISO standard. For regulatory reasons, the items were limited to those classified as “non-critical” parts. Nevertheless, it is worthwhile to consider the work done in this bridging period to better understand what methodologies can be adopted and adapted in the case of future emergencies. This is inclusive of all situations where international supply chains are affected, encompassing emergencies related to public health, natural disasters or climate change. It is important to note that 3D printing may also have great potential in places that are too remote to receive goods shipped from elsewhere and have insufficient resources to support conventional manufacturing industries, such as low-income countries. However, there are practical constraints on what can be successfully 3D-printed, because the spatial resolution is limited, as are the material properties of the resins and filament.

The present paper describes several projects related to the production of bespoke hardware for healthcare applications, beginning with efforts to reverse engineer and 3D-print two specific components of medical hardware. The first was a manifold cover for part of a system that is normally used to deliver oxygen and anaesthetics to patients. This system can be used in emergencies as a ventilator, although with some difficulty (29). The manifold is known to be prone to breakage. A leak valve and leak port were also considered for reverse engineering (30), as these are important components of apparatus used in intensive care of patients with respiratory disease. The leak valve was deemed unsuitable for this project, but attempts were made to reverse engineer the leak port. The manifold and leak port were scanned with optical and/or CT scanning and attempts were made to produce replicas by SLA 3D printing.

Shortages of critical materials were also expected in diagnostic testing laboratories and it was therefore decided to investigate the possibility of reverse-engineering laboratory plasticware needed, using optical scans of the plates, combs and wells used with the standard testing protocol.

This paper also describes the development of new forward engineered designs for a syringe pump lock box used in home-based palliative care. These lock boxes were designed with a view to rapid manufacturing from cut plastic components.

Work on the manifold cover, leak valve, plasticware and syringe pump box provided several insights into the process of engineering projects for emergency situations. Future projects of this kind would be greatly facilitated by a streamlined product lifecycle management (PLM) system (31), which would also represent a step toward the level of manufacturing accountability required by various medical device regulations. PLM software provides a graphical user interface that enables information about products, activities and people to be stored and managed in an organised manner, but it is not widely used outside industry. As the pandemic has seen numerous functions usually performed by industry shifting to the wider community, it is reasonable to argue that PLM methods should be made more accessible. The final section of this paper describes a PLM system developed to manage creation of bespoke hardware for healthcare applications, implemented with the Aras Innovator platform [for another example of the use of Aras see Ref. (32)]. When PLM software is deployed, documents such as standard operating procedures, logs and training records can be embedded in the system. Thus, good document practice can and should be incorporated (33), which will be a requirement if the bespoke hardware is to be submitted for regulatory approval. Our own system would require further testing and development to ensure regulatory compliance.

At the time of writing our hardware and software have not been deployed clinically. The syringe pump lock boxes were intended for testing in a local hospital, but the other hardware has been considered for research purposes only. Any readers of this paper who wish to make use of our findings are responsible for ensuring that they act within the applicable legislation, such as medical device regulations and intellectual property laws.

## Methods

### Optical Scanning

For the purposes of this work, optical scanning was performed using an EinScan Pro (34). This was operated as follows:

- The scanner and turntable were powered up.
- The software was opened, and a new project was created.
- A “non-texture scan” was selected and the appropriate save folder was set.
- The part was placed in the centre of the turntable, fully inside the ring of white circles, where possible.
- The brightness was adjusted until the first signs of saturation were seen. Higher brightness was required for darker objects.
- The scan was conducted with the following settings: HDR = unticked, With Turntable = ticked, Turntable Steps = 16, Align Mode = Turntable Coded Targets.
- Scans were saved and remodelled using approaches specific to each part, as described in the Results section.
- For some transparent parts, chalk spray was used [Montana Chalk Spray Paint Temporary Marking, CH9100 White (35)], as coating items with particles increases the amount of light they reflect (36), without contaminating the objects or adding an offset to the surface.

### CT Scanning

CT scanning was conducted using a Mediso Nanoscan nanoPET/CT scanner (37) (effective resolution ~35-40 micrometres). Scans were acquired in standard semi-circular and helical trajectory, helical and with maximum field of view or medium zoom mode (aiming at achieving better detail). A total of 480 or 720 projections were collected using 50 kVp, 300 ms and 1:1 binning. Images were reconstructed using filtered-back projection, Cosine filter at 100% cutoff, medium voxel size and slice thickness (37 micrometres).

### Post-processing and CAD

To supplement the data provided by optical or CT scanning, for some parts it was also necessary to take manual measurements. Processing and CAD were carried out using an approach that differed depending on which part was being examined, with the software tools SolidWorks, SolidEdge and Autodesk (references given in relevant section).

### 3D Printing

The manifold cover replacement and leak port prototype were produced using a Formlabs Form 2 3D printer (38). A standard clear FormLabs resin was used (39). Following printing, parts were sanded where necessary to remove any sharp edges left after removal of scaffold material and cleaned in IPA to remove residual print resin to prevent further curing that might affect the part shape.

## Case studies

### Manifold Cover for Anaesthesia Machine

**Figure 1:**
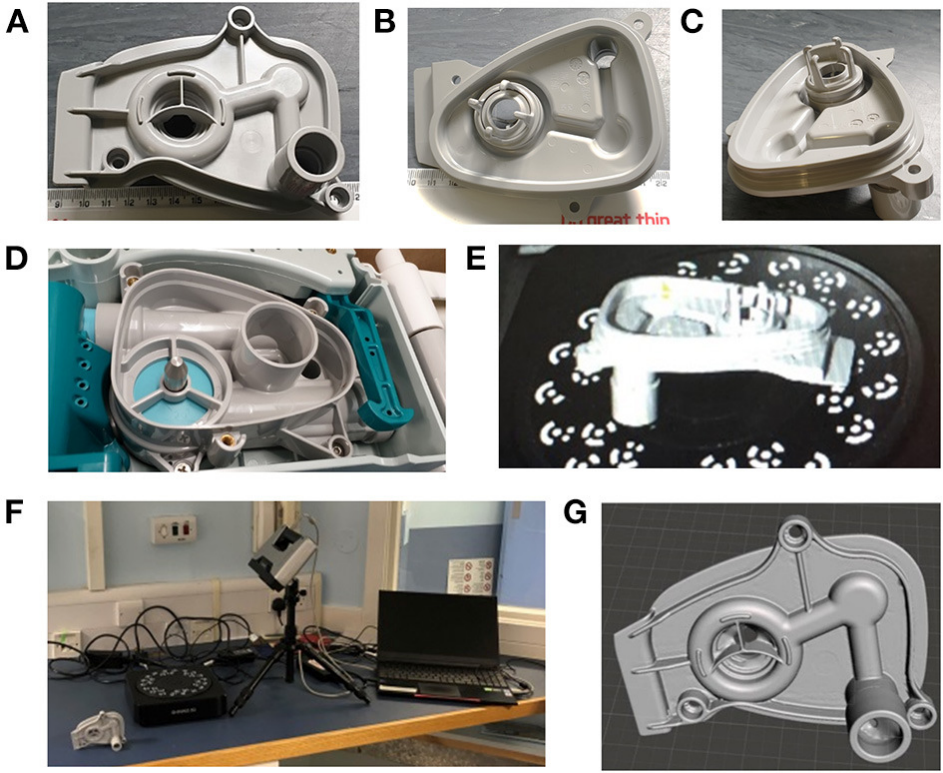
**(A–C)** Different views of the manifold for the Carestation. **(D)** The position into which the manifold fits in the Carestation. **(E)** Depiction of the part in the scanner. **(F)** The Einscan Pro setup used to scan the part. **(G)** The surface mesh after manual adjustments/corrections.

The first case study was the manifold cover of an adjustable pressure limiting (APL) valve circuit from a GE Healthcare Aisys Carestation (Figures 1A–D) (40). Typically, this equipment would be used to deliver oxygen and anaesthetic agents to a patient; however, during the first COVID-19 peak, such anaesthesia machines were utilised as ventilators. At that time, the NHS Lothian Medical Physics team (Edinburgh, UK) identified the manifold cover as being one of the components with an increased risk of damage. It was also noted that the shape of the manifold cover was moderately complex and contained a range of design features common to other parts, including curved edges and surfaces that needed to provide a seal. As such, it was selected as a suitable candidate to assess the feasibility of the reverse engineering process.

**Figure 2:**
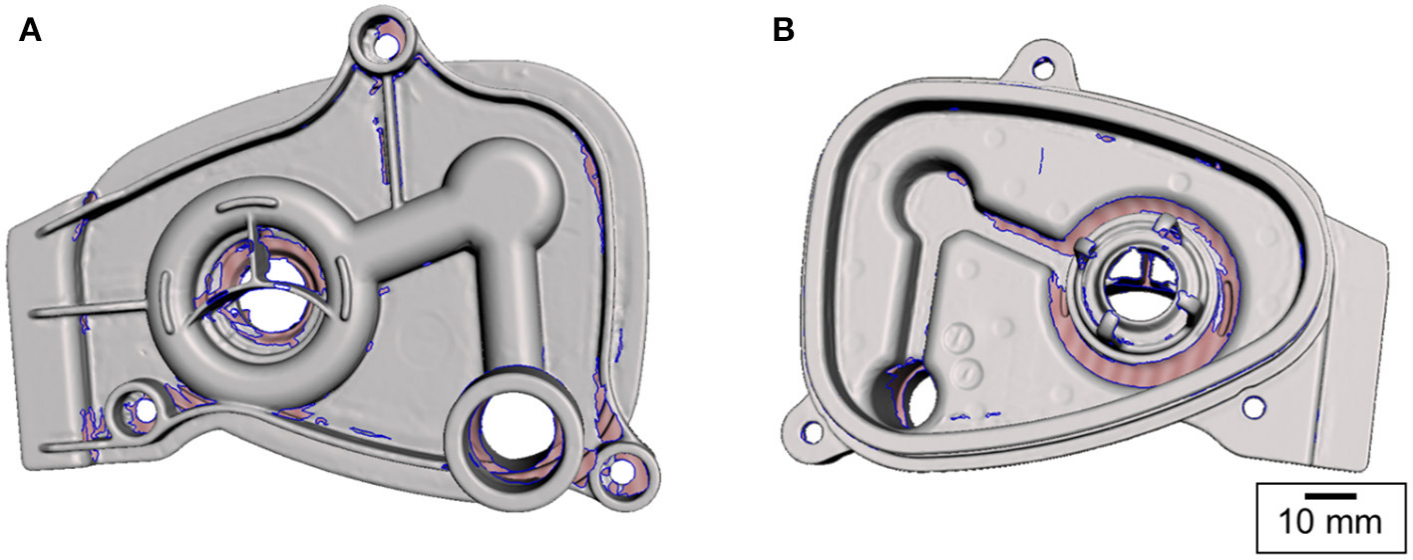
Surface mesh obtained by 3D scanning. The pink-shaded areas (bounded by the blue contours) represent the missing information. The scanner was unable to collect this information due to shadowing [**(A)** top and **(B)** bottom views].

The part was scanned using the Einscan Pro setup with controlled light (Figures 1E,F) and resulting images were manually corrected to obtain the surface mesh shown in Figure 1G. 3D modelling of the part relied heavily on the use of this surface mesh; however, the scan was unable to accurately capture all of the original part details, with the missing information mostly concentrated in the narrow grooves of the manifold port and around the outer sealing surface as marked in Figure 2. The holes in the mesh were caused by the shadowing of these regions. Relatively higher features in the surrounding areas prevented the 3D scanner from accurately capturing the surface topology of the part. This limitation of the 3D scanning process made the modelling more intricate. Hence, to correct the mesh and overcome this problem, mesh post-processing was carried out using Autodesk MeshMixer (41). All information synthesised from the available mesh files was merged into a single representation, capturing features and 3D geometry to create design intent. This required manual repair of the holes within the mesh and removal of extraneous parts. The mesh was then aligned with the world coordinate system, smoothened and optimised for use in a CAD system.

Following the post-processing, the corrected surface mesh was employed as a reference geometry for constructing a parametric CAD model using SolidWorks (42). As the 3D scan data captured only the surface geometry but did not contain any dimensional information, the 3D mesh was calibrated and scaled to accurately represent the manifold cover. This involved measurement of physical dimensions of the part. The bag port diameter and the distances between the manifold cover fixture holes were measured using Vernier callipers. These dimensions were used as the reference values to scale the rest of the mesh. Using the automatic feature recognition functions available within the software package, the reference planes and points were acquired. The semi-manual part creation with a direct mesh referencing approach was chosen as it was found to be more accurate than solely relying on the automated geometry recognition tools. In order to define the solid part features that overlay the mesh body, a series of 2D curves were captured by slicing the mesh and obtaining the cross-sections at a range of heights. These formed the reference contours of the solid part features.

**Figure 3:**
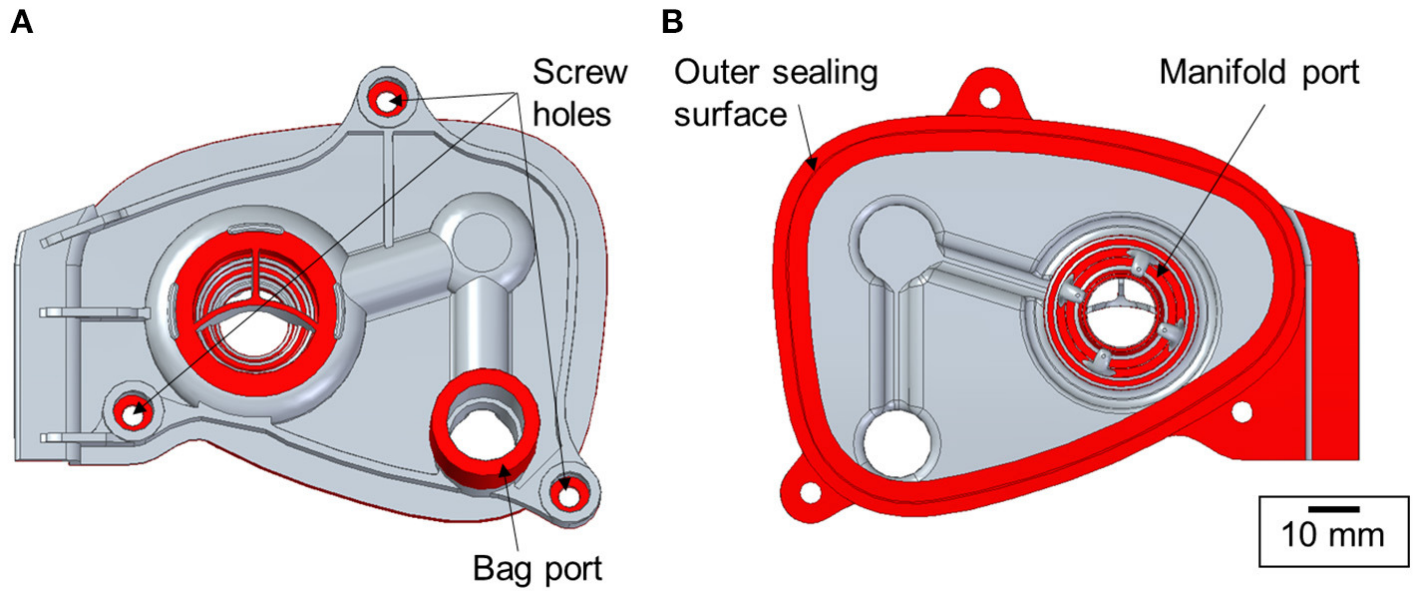
Key functional design features of the manifold cover marked in red [**(A)** top and **(B)** bottom views].

As the turnaround time was an important factor in the process, the manifold cover geometry was partially simplified. The aim was to accurately reproduce only the design features with the critical dimensions such as the sealing surfaces, fixture points and connexion ports. Therefore, the breathing circuit assembly was analysed to evaluate the features of significance. The areas shown in Figure 3, namely, the bag port, manifold port, outer sealing surface and the position of the screw holes, were identified as the key features for the manifold cover to work as a functional replacement part.

Following the parametric CAD modelling, the manifold cover replacement was produced using a FormLabs Form 2 3D printer (38). The SLA method was selected to ensure high 3D print quality, dimensional accuracy and highly isotropic properties. FDM would not have been appropriate as the structures would have been of low quality, with lower strength and poor surface finish.

The original manifold cover was made of polyphenylsulfone, a material of relatively high mechanical strength, good heat resistance and excellent resistance to acids, bases, oxidising agents and most solvents, making it well-suited for many medical applications. With this in mind, a standard clear resin (39) was used for the initial prototyping stage described in this paper. Resin options with higher toughness, thermal or chemical stability (e.g., FormLabs Rigid Resin, Formlabs Tough Resin) could be more suitable for further design iterations.

**Figure 4:**
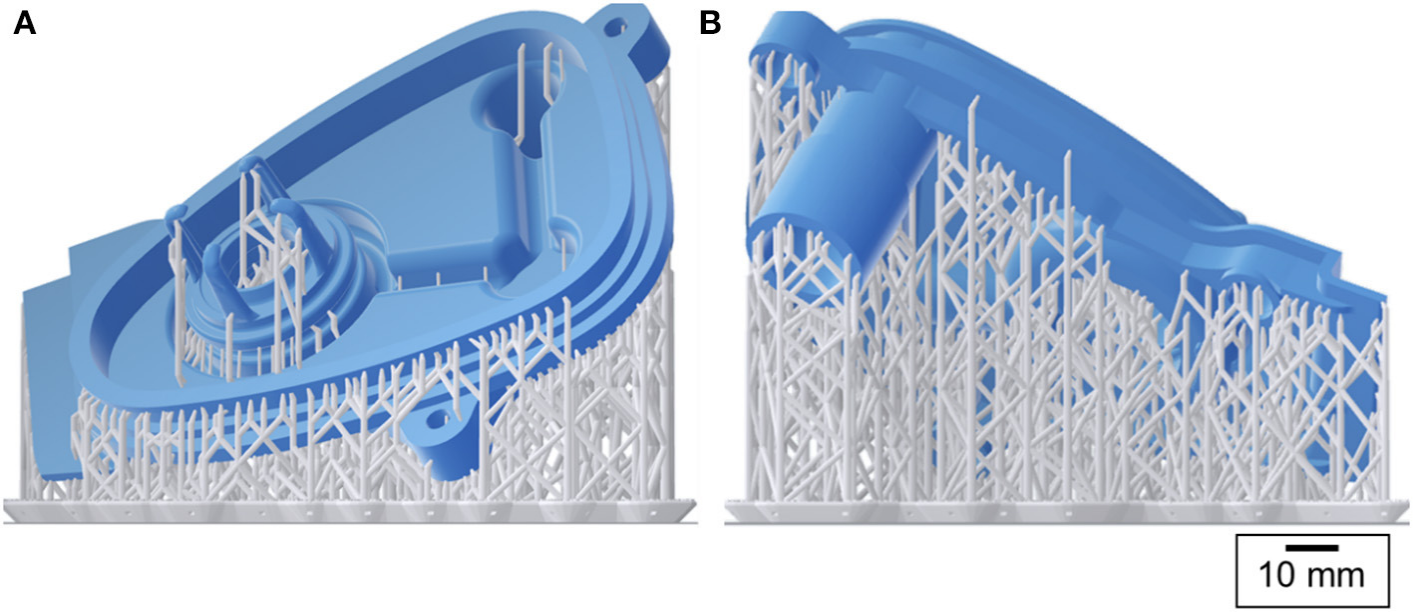
3D printing preview: the manifold cover in blue and the support structures in grey [**(A)** front and **(B)** back views].

The manifold cover was positioned on the build platform so that the required support structures were only attached to the non-functional regions of the part (Figure 4). The printer layer height was set to 0.05 mm and the printing process took 10 h to finish.

During the post-processing of the printed part, the support structures were removed, the part was rinsed in isopropyl alcohol (IPA) and the remaining uncured resin was washed off. No post-curing treatment was applied.

**Figure 5:**
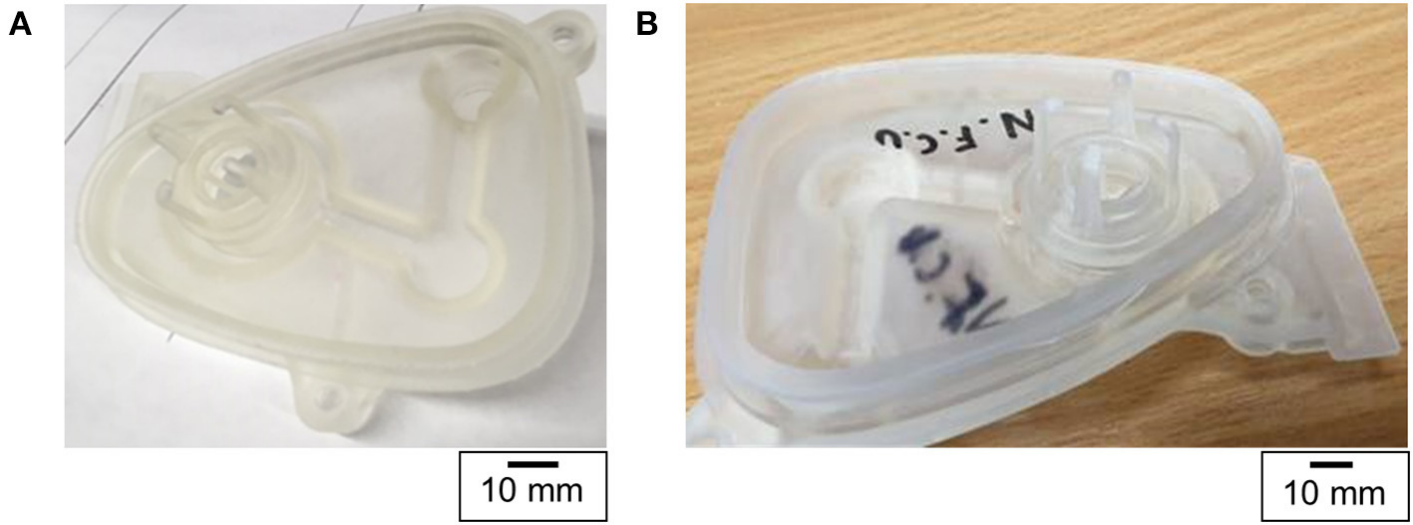
Photograph of **(A)** the first and **(B)** the second manifold cover prototypes made using 3D printing. The letters NFCU stand for Not For Clinical Use.

The evaluation of the first prototype (Figure 5A) revealed that the 3D printed part did not fit the breathing circuit assembly. Key features, namely the diameters of the bag port and the manifold port, were 0.08 and 1.07 mm larger, respectively. The bag port feature was adjusted in the CAD model to compensate for the 3D printing inaccuracies; however, the manifold port was larger due to a design oversight – the O-ring groove on this feature was not identical to the original part. The main reasons for this design inaccuracy were traced to the lack of reliable mesh data, mesh holes in the manifold port region (Figure 2), and absence of physical measurements. To modify the design, a set of physical measurements was taken by the NHS staff on-site.

The second iteration (Figure 5B) of the design showed that the applied changes were suitable and the 3D printed manifold cover fit the assembly. Despite this, a different problem was discovered subsequently – the seal was imperfect and gas leakage occurred. Similar to the first design iteration, the outer surface geometry was solely based on the mesh data and was not reproduced to the required accuracy.

Before the third iteration of the design was completed, the project was stopped as the demand for such systems decreased at the end of the first COVID-19 wave. The experience also suggested that the chosen methods were not ideally suited to this particular part, and without the know-how of the original manufacturer it would be practically impossible to guarantee long-term satisfactory performance of the part. Furthermore, the legal and regulatory situation presented insurmountable challenges, and it was not clear whether the part could have been used even if its performance had been appropriately validated. The project is still highly instructive, as it reveals the strengths and limitations of the processes used, and some of the methods could be applied more successfully to other parts.

Due to the remote working arrangements caused by the COVID-19 pandemic, the design process was highly challenging. The manifold cover had to be designed by an individual who did not have physical access to the original part. Therefore, the design heavily relied on the 3D scan data while a few design decisions were based on the information provided in the technical reference maintenance manual (40). The physical measurements of the original part remain a crucial aspect of any reverse engineering process as the data collected using 3D scanning approaches often suffer from shadowing and mesh distortion. Accurate reference measurements are vital during the mesh editing stage and can highly reduce the number of design iterations.

### Leak Valve and Leak Port

**Figure 6:**
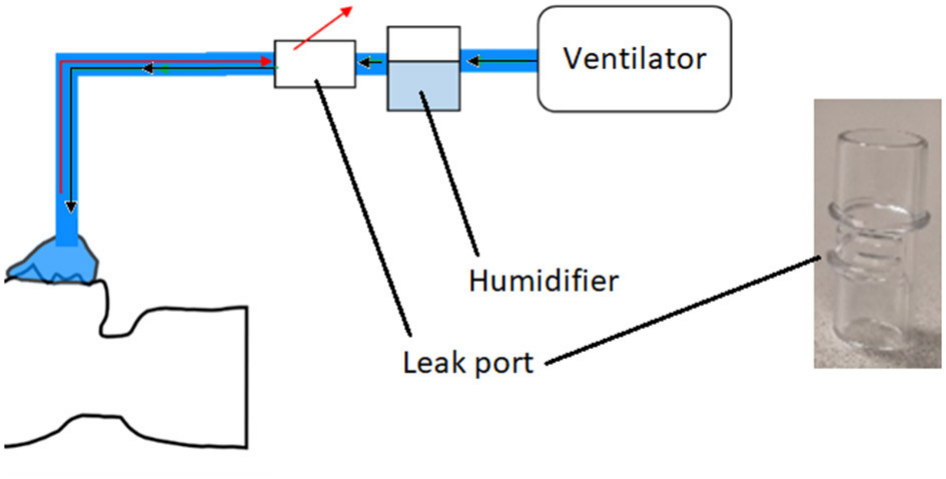
Schematic of non-invasive ventilation system. Black arrows represent air going to the patient, and red arrows are exhaled gas. Also shown is a photograph of the original ResMed leak port.

During the COVID-19 pandemic, ventilation was used extensively as a treatment for severe respiratory symptoms. Non-invasive ventilation (NIV) involves the provision of air via a mask and is commonly used for treatment of COVID patients (43). In this project, components of NIV systems were considered as candidates for reverse engineering, as many hospitals were experiencing supply shortages. Two specific components were considered, both originally supplied by the manufacturer ResMed: a leak valve and a leak port. The former contains an anti-asphyxia valve, which is designed to protect the patient if the air supply fails. Leak valves were in short supply during the pandemic, and ResMed released guidance on the use of the leak port in place of the leak valve in emergency situations (44), noting the inherent risks. The leak valve/port was to be installed in the air line between the ventilator and the patient (Figure 6). As a result of the aforementioned supply shortages, NHS staff anticipated a potential need for a back-up option should both parts become unavailable. The reverse engineering and production of leak valves and leak ports was therefore chosen as the second case study for this project.

Initially, optical scans of the original parts were obtained using the Einscan Pro system; however, due to the transparency of the parts the image quality was too poor to use. To overcome this, the parts were coated in a chalk spray. The part was scanned again, and the scan was processed using Einscan software that allowed a point cloud to be converted to a 3D model, before being exported in STL format. Although the chalk spray made the part opaque enough to scan using the Einscan Pro system, it was only able to capture the larger features of the part. As a result the leak port was then scanned using a Mediso Nanoscan CT scanner (effective resolution 35–40 μm). Scans were acquired in standard, helical and medium zoom mode.

During the initial evaluation of the scanned parts, it was determined that the leak valve would be too complex to reverse engineer and produce using the techniques available at the time. This was due to it being made of multiple components and materials, i.e., it incorporated an anti-asphyxiation valve. As such, the remainder of this case study focused solely on replicating the leak port.

**Figure 7:**
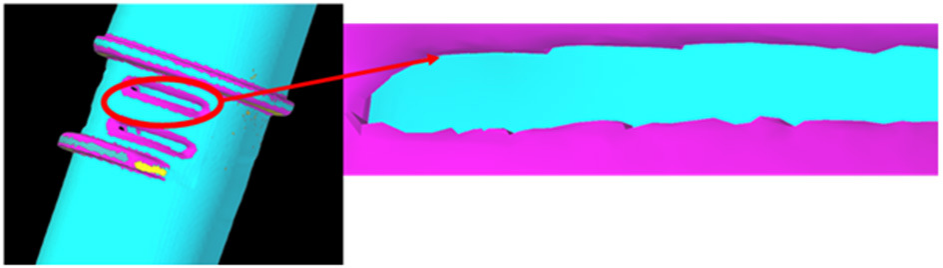
CT scan of the leak port. Due to limitations of the scanning equipment, the resolution of the slot features was poor.

CT scans were processed using 3D Slicer software (45), this allowed images to be segmented, and converted to a 3D model, for export as an STL file. The optical and CT models were imported into Solid Edge (46), where reverse engineering techniques (similar to those described in the previous section) were employed to fit surfaces to the STL that allowed measuring of the features and for a new 3D model to be produced for printing. Although larger symmetrical features were readily duplicated, the main challenge was matching the slot features on the front of the port. These slots were very small, and, as the parts were scanned using medical grade CT, the resolution was relatively low compared to industrial standards (Figure 7). This meant that the edges appeared jagged and could not be easily copied from the scans. As an alternative, Vernier callipers were used for additional measurements.

**Figure 8:**
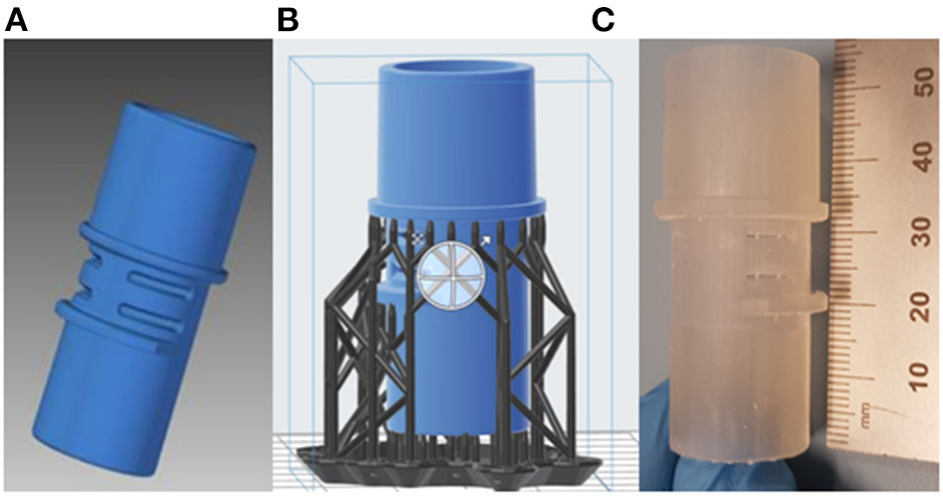
**(A)** Final CAD model of the leak port; **(B)** Pre-processing of leak port, with support structures; **(C)** Printed leak port.

The leak port consisted of a series of symmetrical features, but it was not possible to determine their dimensional tolerances for manufacture. When these features were measured during the CAD phase it became apparent that they need to be accounted for in the final model. As a result, measurements were taken from multiple areas, and averages taken. An assumption was made that the design intent was for these features to be as symmetrical as possible, with the measured bounds assumed to provide a maximum and minimum allowable deviation. The final model (Figure 8A) was made transparent and overlaid with the original scan file to identify regions that may have large deviation.

The final part was printed using the same FormLabs Form 2 printer as described in the previous section, with a print time of about 5.5 h. Due to the overhanging features on the part, additional lattice supports were applied in pre-processing, as seen in Figure 8B. These were removed and sanded down after printing was finished. The final part was printed using the same clear resin described in the previous section, and is shown in Figure 8C. Following printing parts were cleaned in IPA to remove residual print resin. Before real use, parts would need to undergo sterilisation to ensure parts are clean and any unwanted particles, chemicals or bacteria have been removed.

When evaluating the replicated leak port, it was important to discuss a number of key points that were identified during the reverse engineering process in regard to efficacy and patient safety. Although the final model matched the scanned data well, only one port was available at the time for assessment. As a result, there was a reduced data set with regard to measurements, which had the potential to effect the accuracy of the replicated part, and possibly effect how well it interfaces with the ventilation system, and/or how well it functions for its intended purpose. Furthermore, as with the manifold cover, there was also the possibility that issues with accuracy could be exacerbated by distortions during scanning and manufacturing, and/or incorrect assumptions made during the design phase. Therefore, validation that the part fits and functions as required would be necessary before the part could be used. It would also be essential to evaluate the best material for replicating parts. As mentioned in the previous section, there were a few medical grade polymers were considered for resin printing; however there was insufficient scope in the project to explore this further. For future work, the selected material must be biocompatible, strong enough and capable of being sterilised before use without detrimental changes to the final part.

It is interesting to speculate about alternative methods for addressing a shortage of leak ports. It may be possible to use components intended for other purposes, with or without modification. For example, there may be connectors of similar dimensions but lacking slits, which could be modified by addition of the necessary holes. This process would need to be carried out carefully to avoid splitting the connector.

### Plasticware for COVID-19 Testing

At the outset of the pandemic, it became apparent that there would be the need for widespread diagnostic testing. Such tests were developed rapidly, with both commercial and more cost-effective solutions now widely available (47, 48). The standard and most sensitive test is based on the detection of ribonucleic acid (RNA) from SARS-CoV-2, the coronavirus that causes COVID-19, using reverse transcription quantitative polymerase chain reaction (RT-qPCR). The first step in this process is the isolation of RNA from patient samples, the efficiency of which is greatly improved by the use of automation. One widely used procedure in the UK (49) employs the KingFisher™ lab automation platform [developed by Thermofisher Scientific™ (50)]. The KingFisher™ system uses 96-well plates with a corresponding tip comb, both made from polypropylene. To prevent contamination between samples and ensure accurate test results, new plates for both sample wells and tip combs are used for each run. This requirement, combined with the rapidly increased demand of RT-qPCR testing, and reduced capacity of production and delivery due to lockdown, led to concerns that shortages in plasticware could hinder mass scale testing (51).

In the laboratory, liquid samples are placed in deep wells in a 96-well plate. The corresponding tip comb is attached to a movable head within the KingFisher™ system. The tip comb is lowered into the 96-well plate to add or remove particles from the samples. Magnetic rods within the KingFisher head are inserted into the tip comb when there is a need to attract magnetic beads to the tips (The manufacturer supplies a video: https://www.youtube.com/watch?v=cace7rRTIww).

If the original manufacturer was unable to meet the demand for plasticware, it was hypothesised that production of KingFisher™ compatible plates could be outsourced to other institutions, such as universities, or other industrial producers of plastic products (including for instance manufacturers of disposable cutlery and similar items). The designs were proprietary, and it was uncertain if permission would be granted to begin 3rd party production should the need have arisen. As the pace of the pandemic increased, it was decided that, as a 3rd case study within this project, 3D designs of the existing plates/combs should be prepared in the event that reverse-engineered plasticware would be needed at short notice and legal clearance would be given for their use. It was also unclear what legal clearance would be needed, as it appears that the KingFisher system was originally covered under US patent 6448092 (52), which expired in 2015, but there may be other applicable patents that are still in force.

In contrast to parts such as the leak port, in this case the shape of the plates and combs is absolutely critical to their function and faithful reproduction would be required to ensure compatibility. Optical scanning with a chalk spray was attempted for the laboratory plasticware, but the quality of the scans was insufficient. CT scans of the deep well, tip comb and holder plastic plates were performed using a Mediso nanoPET/CT scanner, as described above. These scans generated 3D models of each plate, but they did not cover the entirety of each plate. There were gaps in the model, and as the plate was thin the plastic was prone to bending. This meant that the CT scans were not suitable for direct replication, but, instead, were used as references to model the plates in combination with manual measurements.

**Figure 9:**
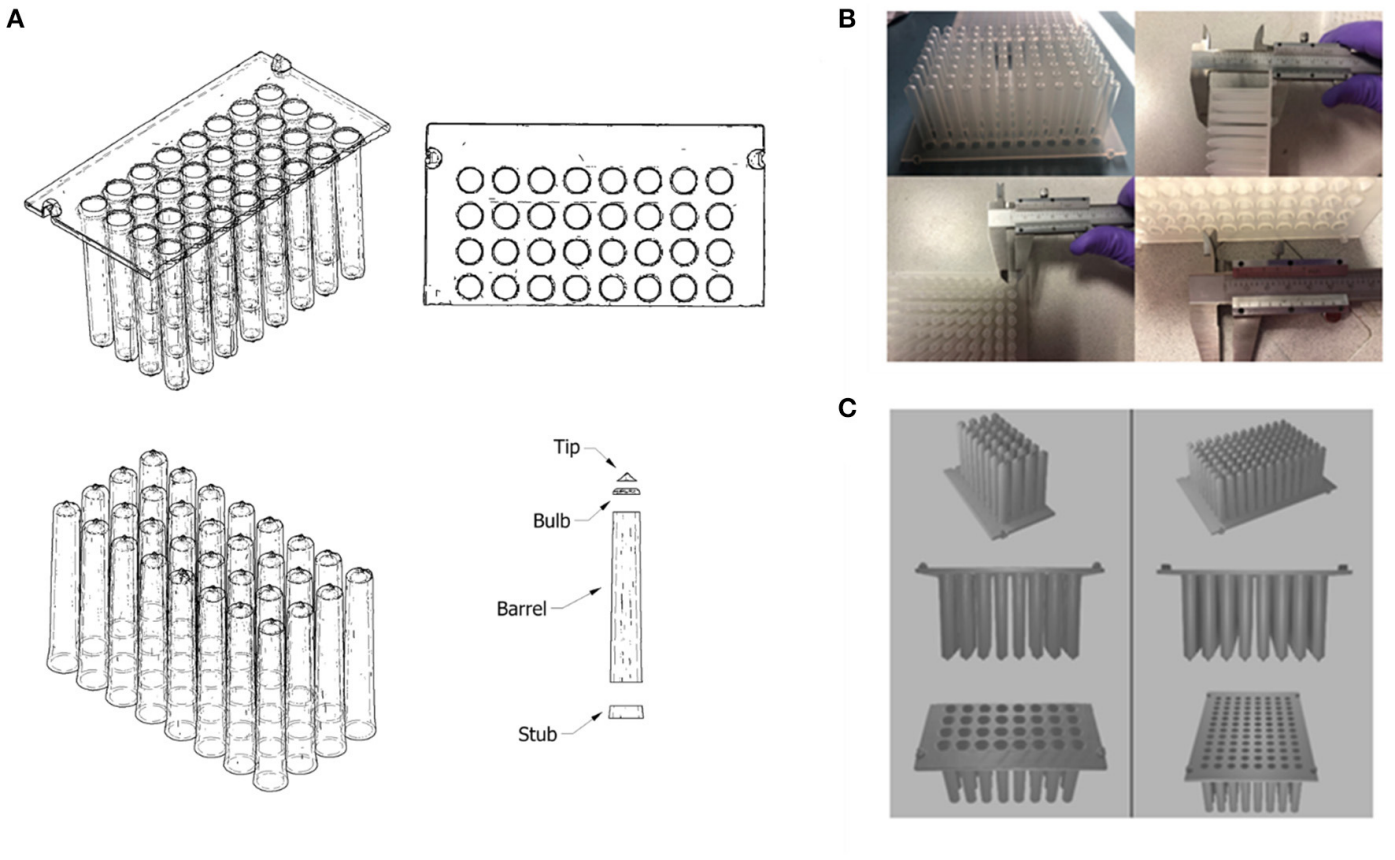
**(A)** Autodesk Inventor model (wireframe with hidden edges) of a CT scanned 3D model of part of a Kingfisher™ comb plate. The model was split into distinct parts to allow for the measurement of its dimensions; **(B)** Manual measurements of the original Kingfisher™ comb plate using Vernier callipers. **(C)** Final 3D model of reverse engineered Kingfisher™ compatible plate, which was rendered using Blender.

To test this approach, the tip comb plate was focused on as an example. Autodesk Inventor (53) was used to generate sketches of the CT scans that would allow for dimensions to be measured, such as the diameters of holes, the thicknesses of walls, and the grid spacing for a 96-well configuration (Figure 9A). These sketches accommodated measurements of the complex structure, which would otherwise be difficult with manual measurements. It also helped to account for variances in dimensions produced by the CT scanner and curvature of the original plastic part. Measurements were taken at multiple points and averaged to account for variances in the CT scan. Measurements were also cross-referenced against manual measurements taken with Vernier callipers (Figure 9B).

To simplify the production of a 3D structure, the design was split into 2 parts: the base plate; and the teeth of the combs. The base plate was a simple flat plate that featured holes in a 96-well grid configuration, and 4 raised fittings on the outer edge that allow for mounting in the Kingfisher system. The individual teeth were split into 4 parts (dubbed “stub,” “barrel,” “bulb” and “tip”) to measure the changing diameter of the inside of the tip at each stage and allow the varying angle of the outside of the combs to be recreated as closely as possible. Once a tooth design was produced, it was duplicated and aligned with the 96 holes on the base plate and the two parts were merged to produce one final 3D file (Figure 9C) using Blender (54).

The dimensions of the inner, hollow sections of each comb were of particular importance, as magnetic rods will be moved in and out automatically by the Kingfisher system machinery. If the inner chamber were too narrow or too short, this could result in damage to the machine or malfunction during operation. It was also important to maintain the thickness of the walls. As the magnetic rods within the comb are used to attract magnetic beads in solution, increasing the thickness of the walls could have reduced the efficacy of the process, affecting the performance of the testing protocol. If the walls were too thin, the part could have been too weak. In this case, the part might have been vulnerable to breakage during operation, potentially contaminating the magnetic rods. The slope of protruding features would be important not only to ensure that all the parts fitted together as required, but to ensure effective removal from a mould during mass production.

Fortunately, these designs were never needed. It is important to note that for mass production, 3D printing would not be suitable due to the long print times needed to produce a single plate (at least several hours per plate). Nevertheless, with a variety of temperature and force resistant resins and filaments available, 3D printing could potentially be used to rapid prototype mould tools for conventional manufacturing processes which are better suited to mass production e.g., injection moulding or vacuum forming (55, 56). Factors such as sterility and potential contamination from pyrogens, RNases, and DNases would still need to be addressed. As 3D printing was not a suitable technique for mass production, prototypes were not tested, but the design is believed to be sufficiently accurate for use.

While the design work was being carried out, some laboratory tests were performed that suggested plasticware could be cleaned and re-used. While this is contrary to standard molecular biology practice, it is worth noting that this procedure could be adopted in case of future shortages. This approach would potentially be superior to the use of reverse-engineered plasticware, and would also be more sustainable, leading to generation of less plastic waste. Other solutions could potentially be found, further discussion of which is beyond the scope of the present work.

### Syringe Pump Lock Box

**Figure 10:**
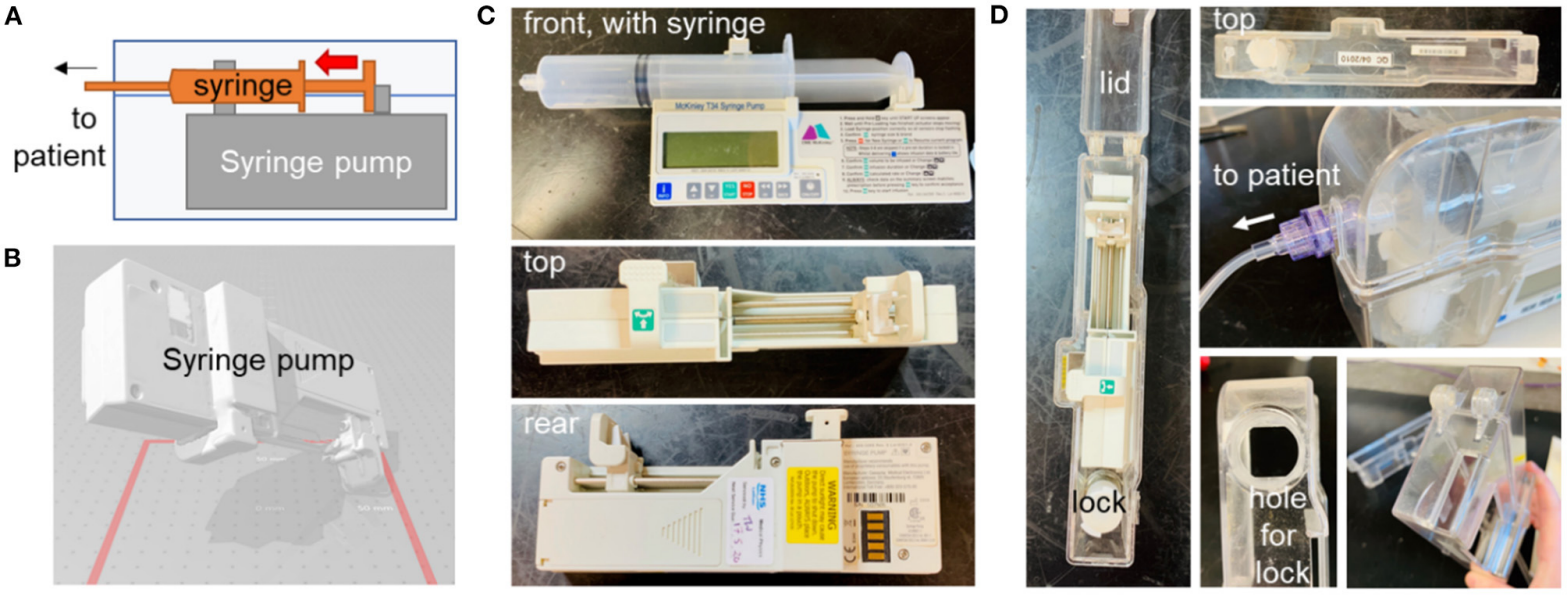
Syringe pump and original lockbox. **(A)** Schematic diagram showing syringe pump with syringe, enclosed in lock-box. **(B)** Optical scan of pump, to assist with dimensioning of new box. Photographs of syringe pump **(C)** and existing lock-box **(D)**, various angles.

During the pandemic, much attention was focused on ventilators and related systems, but there has been a massive impact on all aspects of health and social care, changing the way in which healthcare is delivered. For example, as the COVID-19 pandemic placed more pressure on hospitals, more patients were expected to undergo palliative care at home. This often involves the administration of controlled painkillers via syringe pump drivers, which administer medication such as morphine subcutaneously in a continuous manner over an extended period (57) (Figures 10A–C). To prevent tampering with the syringe pump during operation (for instance by carers), the syringe pump is secured within a lockbox (Figure 10D). These lock boxes are prone to breakage, particularly around the moving parts (hinge and locking mechanism). A potential shortage was identified due to increased demand and potential lack of availability from the supplier, BD Healthcare. It was therefore decided to investigate the feasibility of producing an alternative syringe pump lock box.

**Figure 11:**
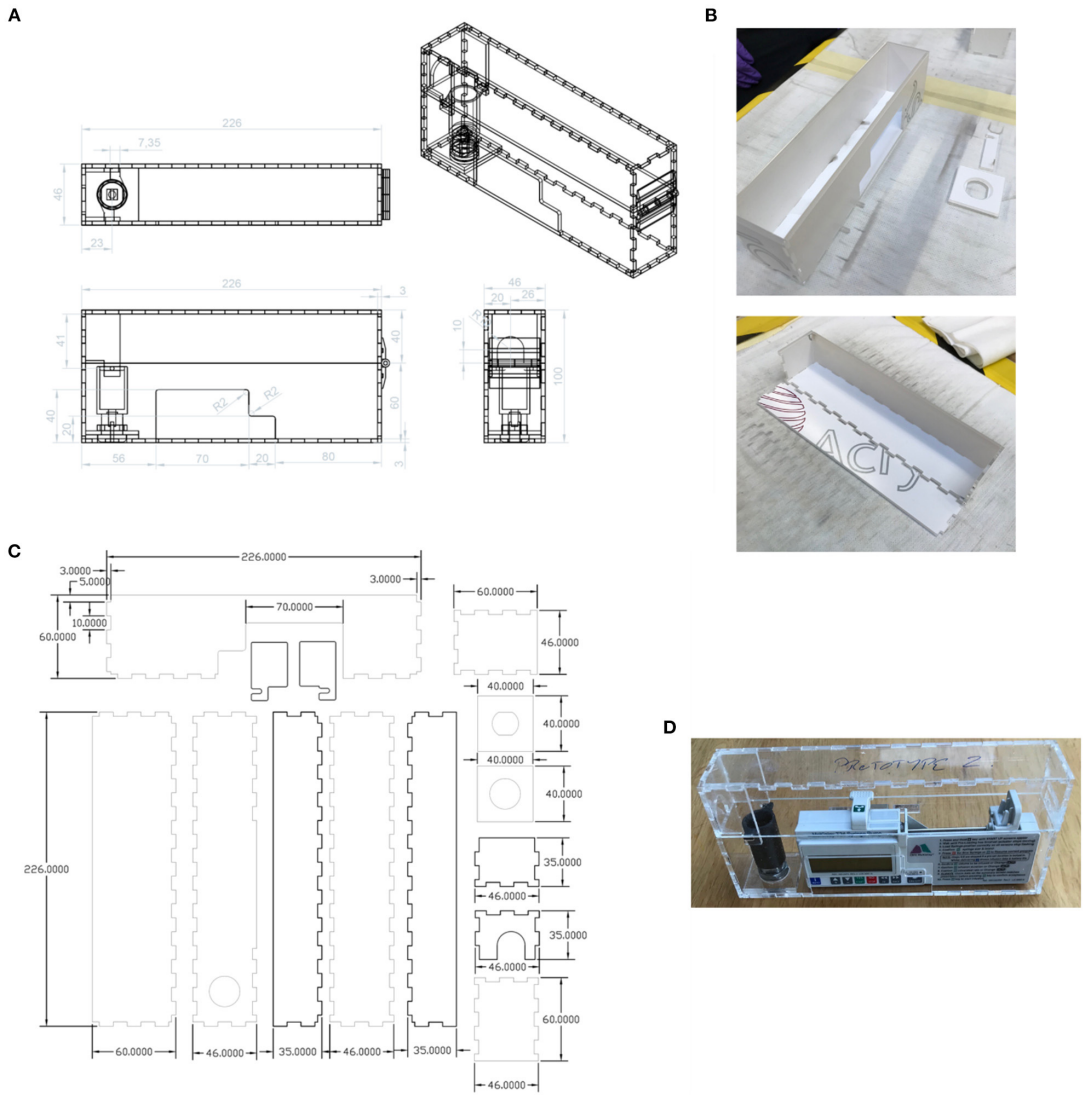
**(A)** New design of lock box, made using laser-cut plastic parts. Units: mm. **(B)** Assembly of the lock box. **(C)** The parts to be laser-cut from the plastic sheet. Units: mm. **(D)** The assembled prototype (without edging).

The new lock box was required to hold the pump securely, with cut-outs to enable access to the display, control panel, and battery access panel. An opening was required for the drug delivery line attached to the syringe. The lock box would need to be robust and resistant to damage if dropped. The lockbox was required to house a pump carrying a syringe as large as 50 mL. Cleaning of the box would typically be performed with detergent wipes. As the box would need to be produced quickly in a workshop (such as that in a university) at low cost and a small number of units would be required, it was to be made from laser-cut plastic components rather than by injection moulding or other similar method. The new box was to be compatible with the barrel locks of the original boxes, because the locks are less susceptible to breakage than the box itself. A new box was designed to meet this specification (Figure 11), with a 3D-printed lock mechanism to incorporate the original barrel within the new design. As the T34 syringe pump is designed to be portable, the lockbox does not need design features that would allow it to be secured to a solid fixture.

Prototypes of the new box were made from 3 mm thick Extruded Acrylic. The laser cutter used was an HPC 150W CO_2_ laser cutter (58), The settings used were 100% power at 12 m/min. The lock spindle, designed to fit existing supplied lock barrels, was 3D Printed in ABS on a Fortus 250 mc printer (59). The plastic sheet parts were cleaned with IPA before assembly. Interlocking panels were bonded together using adhesive Tensol 12 (60).

The new lock box is designed to hold the pump securely, preventing lateral motion. In clinical use, the box is often subjected to impacts, for example when falling from a patient's bed to the floor. Consequently, protective edging is required. A medical grade polymer edging strip silicone L section PC60, supplied by Advanced Materials Ltd (61) was tested, but the finish was found to be unsatisfactory and a 3D-printed frame was produced instead. The new box is believed to be suitable for clinical use but at the time of writing it has not been deployed.

### PLM

**Figure 12:**
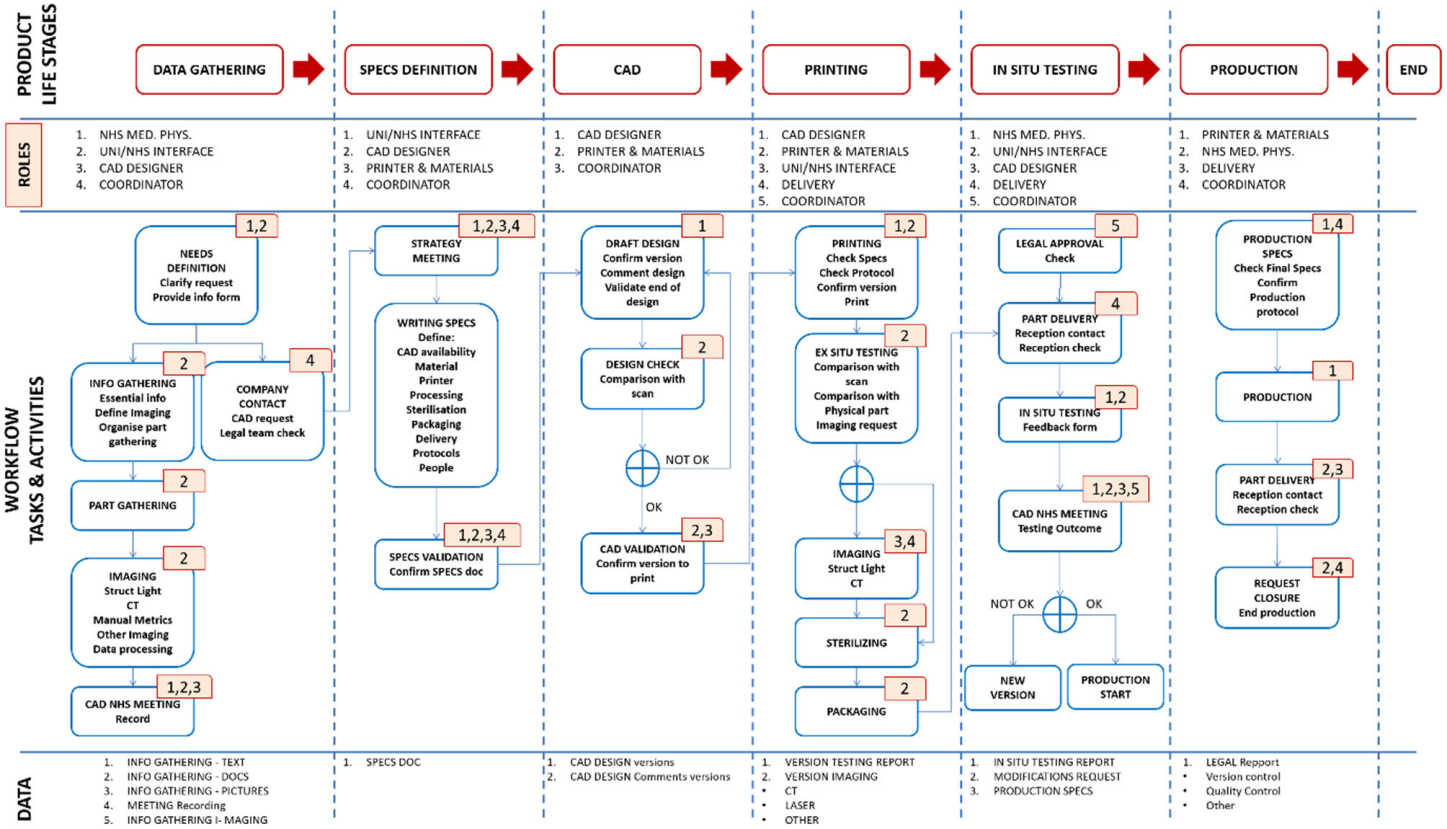
Schematic diagram of the Aras PLM tool for the production of bespoke medical equipment. The first row highlights the product life stages. The second row identifies the personnel roles at each stage. The third row shows the workflow of tasks and activities. The roles associated with each task or activity are identified by the corresponding numbers (top right corner of each task box) e.g., the first task in the “data gathering” stage has numbers 1 and 2, meaning it involves NHS Medical Physics personnel, and personnel working at the interface between the NHS and the engineering organisation. The fourth row outlines the data output.

The experience gathered from engineering the manifold, leak port, plasticware and lock box was used to produce a detailed Product Lifecycle Management (PLM) tool with appropriate workflows to coordinate the production of bespoke medical equipment. This tool was implemented using Aras Innovator – a software platform for complete, end-to-end PLM. Aras had the capability to cover various elements of PLM, including requirements, engineering, manufacturing, and operation. During this project, PLM tools, such as the one shown in Figure 12, led to the production of good practice documents reporting on decisions and allowing for validation and traceability. The PLM workflows refer to the “University of Edinburgh,” but this is a placeholder for any engineering organisation that could be helping to provide emergency manufacturing support to a local hospital.

The workflow, shown in Figure 12, was separated into clear product life stages, which could be validated by the production of signed technical documents that provided process clarity and traceability. These stages included:

1. Data Gathering
  - Definition and technical specifications of the part by NHS staff (i.e., documents, pictures, recorded meeting with the CAD designer).
  - Where required: part scanning using appropriate imaging modalities (e.g., optical scanning with/without chalk spray, CT scanning and image refinement/correction).
  - Production of a user requirement specification (URS) document.
  - If situation permits, designer should observe the situation in which the part is to be used or be provided with alternative option such as a video.
2. Specifications Definition
  - Meeting organised by the technical production team and the coordinator to assess the gathered information and to define a production strategy.
  - Production of a technical specifications fill in form (designated by the abbreviation FRM; there would need to be a form for each key step, as indicated here).
3. Computer-Aided-Design (CAD)
  - Production of designs.
  - Comparison with the part scans (where applicable) and assessment by production technician.
  - Production of a CAD validation fill in form (FRM).
4. 3D Printing and *Ex Situ* Validation
  - Production of part (by e.g., 3D printing) using the validated CAD design, SOPs and the technical specifications fill in form FRM.
  - Where applicable: comparison of the original part and its replicate – using both manual measurement and additional imaging (optical or CT scanning).
  - (Contingent on achieving acceptable part quality for *in-situ* validation) Sterilisation and packaging as defined by the technical specification fill in form.
  - Production of a 3D printing fill in form (FRM) or equivalent for other production technique
5. *In situ* validation
  - Confirmation of legal approval prior to any assessment/use.
  - Delivery of part and *in-situ* assessment by the NHS Medical Physics team.
  - Meeting organised with CAD designer to discuss the results of the *in-situ* assessment and identify any further design iterations.
  - Decision made on part redesign or initiation of production.
6. Production
  - Confirmation and approval of final technical specifications.
  - Creation of a production SOP.
  - Initiation, and subsequent continuation, of production and delivery process to reach final production numbers.

A blank user requirement specification document, SOP and fill-in form are provided in the Supplementary Information, together with a “coordinator's manual.” Additional documents would be required to record some aspects of the project such as the training of personnel.

For each new part to be produced, five roles within the PLM had to be assigned:

1. A part production coordinator
2. An NHS Medical Physics contact person
3. A CAD designer
4. A 3D printer and material contact
5. A delivery contact

Importantly, these roles were part-specific so individual roles could change between production pipelines for different parts. Also, the coordinator had the option to replace anybody in the pipeline if required. This would be crucial for emergency production pipelines where individuals might have had multiple external constraints leading to an important turnover, or simply in case someone needed to be replaced (e.g., one individual getting COVID-19). The PLM workflow facilitates tight collaboration between clinical staff and designers, with an iterative process that can enable a rapid turnaround if fully implemented.

**Figure 13:**
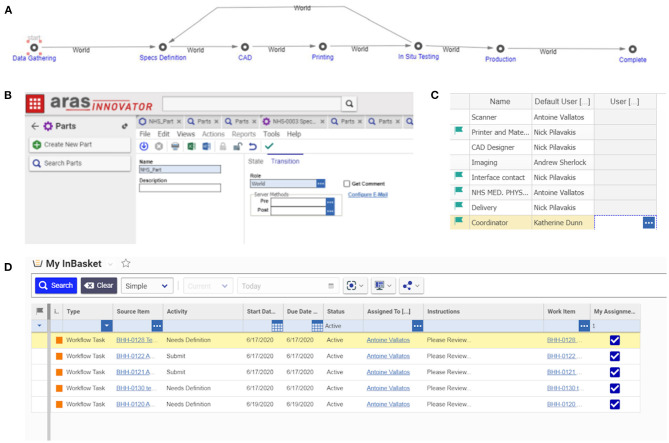
PLM implementation into Aras Innovator – a platform for the coordination of bespoke medical equipment: **(A)** the PLM stages in Aras, each stage requires a number of tasks to be accomplished and documents to be produced; **(B)** each new job begins with the start of a new PLM life cycle; **(C)** roles are assigned to contacts; **(D)** the software advances through the life stages informing people of the tasks they have to accomplish automatically, either on the online platform or by email.

The Aras Innovator software (see Figure 13) facilitated all these steps by providing a visual interface, PLM overview, reminders for personnel on required actions, good practice documents, and project history. The implemented PLM tool included up-to-date template documents for validating each step while it recorded all changes for future reference. For loop processes involving several iterations, a versioning system for good practice documents was integrated to the software. In the event of going to full production, these various features would have been crucial for achieving part certification, as every step (design, printing, assessment) would have needed validation and reporting in appropriate documents.

Although full production did not occur within the lifespan of this project, and further development would be needed to ensure regulatory compliance (in line with applicable ISO standards on medical devices, testing and quality management systems), the PLM tool developed here could act as a valuable starting point for creation of a tool to be used in future emergency scenarios where the movement of people and supplies are restricted, and distributed manufacturing becomes a crucial alternative. Importantly, PLM tools such as Aras enable users to make many modifications and adjust the pipeline for alternative workflows (for example, using techniques other than 3D printing). Clearly defined work packages and documented product life stages are key in order to achieve robustness and fluidity of the workflow while ensuring accountability. Software platforms other than Aras could be used and lessons learned here would be transferable to other systems.

## Critical Evaluation of Prototypes

**Figure 14:**
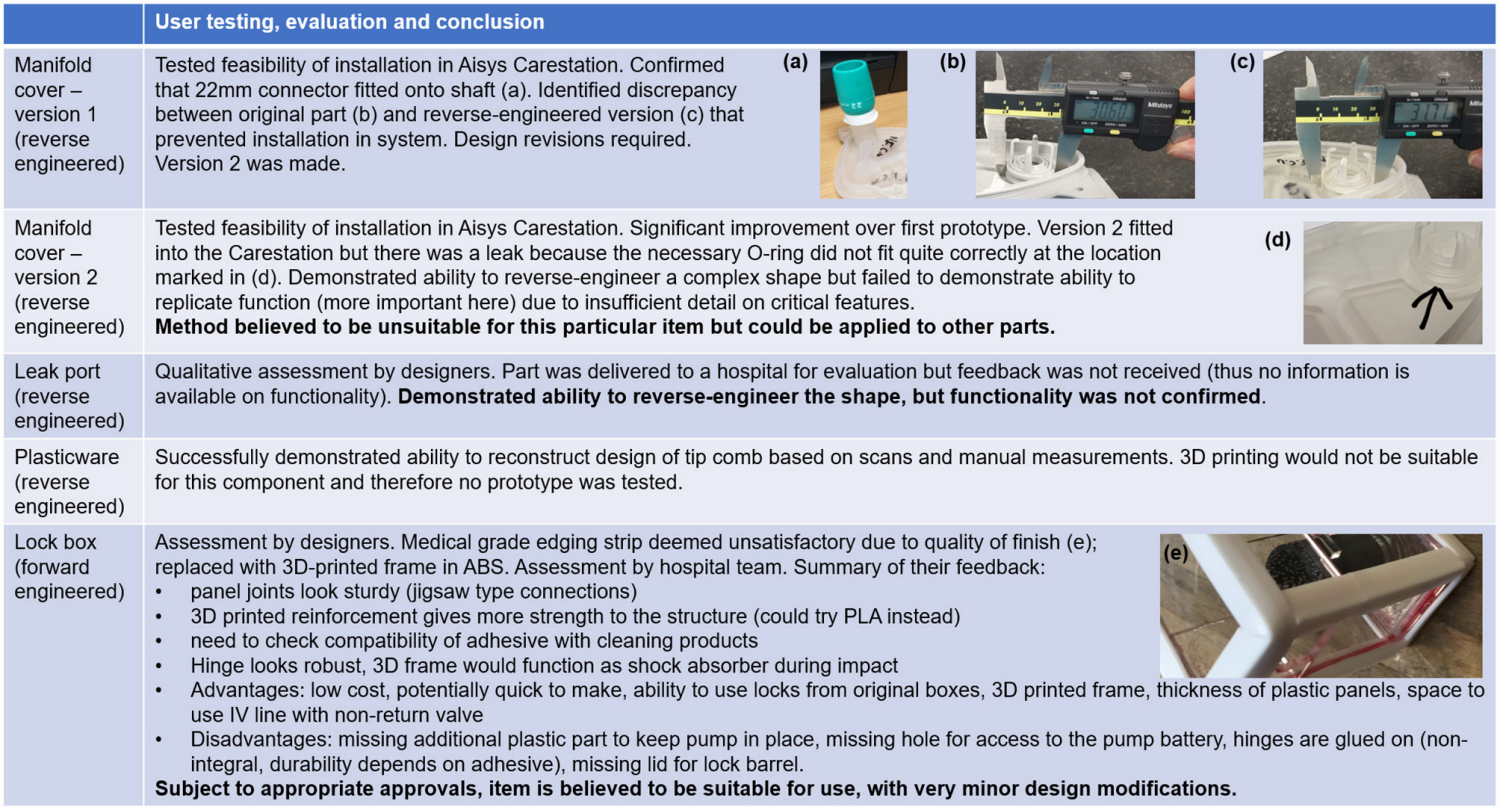
Critical evaluation of components. Table shows user testing, evaluation and conclusion, with embedded photographs illustrating areas for improvement for each prototype examined. **(a)** 22 mm connector fitted onto shaft of first version of reverse engineered manifold cover **(b)** original manifold cover, with critical dimension shown **(c)** first version of reverse engineered manifold cover, with discrepancy shown **(d)** source of leak in version 2 of the reverse engineered manifold cover **(e)** unsatisfactory edging on syringe pump lock box.

As discussed above, the second version of the reverse-engineered manifold cover could be installed in the Carestation but was not fit for purpose due to a leak in the system (Figure 14). In the case of the leak port, the approaches adopted did enable the physical form of the part to be replicated with reasonable accuracy, but the function of the part could potentially have been reproduced by a part with shape differences. In contrast, accurate reproduction of the shape of the tip comb would be essential to ensure appropriate function, and the design was believed to be sufficiently accurate for use (Figure 14). It was not tested as 3D printing would not be suitable and more appropriate manufacturing methods were not available.

The lock box was the most successful of the four hardware projects. Based on feedback from the hospital team, it is believed to be suitable for use, subject to appropriate approvals and very minor design modifications (Figure 14). The lock box was designed by forward engineering rather than reverse engineering. In many respects, its design was less constrained than that of the other components. Its performance was also easier to assess, as defects would be more readily apparent. The manufacturing method chosen would be suited only to low-volume production, as with the manifold cover and leak port. The lock box represents an example of forward engineering a new solution rather than reverse engineering. Other alternatives to reverse engineering include re-using old parts (reconditioned if required) and identifying other components that could be modified to perform a similar task. Re-use was noted as a viable approach for addressing plasticware shortages, and the possibility of re-purposing other components was noted in the context of the leak port. The manifold cover is probably the most problematic of the parts examined here, as re-use is unlikely to be an option if the part is broken, the part is so specialised that other components could not replace it, and reverse engineering has limitations, as has been shown.

## Conclusions

Due to the evolution of circumstances, the design process for some of the items was not completed. Our project was driven by real-world needs, and as the situation changed, the demand for parts such as the manifold, leak port and improvised plasticware faded. Consequently, decisions were not taken on aspects such as the final material choice, the sterilisation procedure and the final form of the designs. However, there are numerous insights that can be extracted.

Most of the activities covered were performed in the early stages of the pandemic, during lockdown, when COVID-safe working practice were very new. This presented some difficulties in terms of the movement of parts and personnel. For instance, in the case of the manifold, the CAD work was carried out by an individual who had never had access to the actual part itself. This experience underlined the importance of the data gathering and specification stages of the development pipeline, and this was captured in the PLM system developed. Involvement of end users in the design process is important, and design should be iterative, with ongoing dialogue between designers and front-line clinical professionals. Excellent communication and good documentation would be essential for large scale deployment of adaptive manufacturing for future emergency scenarios. The use of PLM would ensure that the workflows are well-suited to the ultimate objective, whether that is the manufacture of many items, custom production of a single part, or simply generating data to inform important decisions.

The project indicated that reverse engineering is not necessarily suitable for all components, although it did confirm that complex shapes such as the manifold or leak port could be reproduced to some extent by reverse engineering and rapid prototyping. There were numerous challenges, both technical and otherwise, some of which remain unsolved. Some features were difficult to capture and reproduce, examples being the O-ring of the manifold and the slots in the leak port. Sometimes the reconstruction of the geometry can be facilitated by the removal of unnecessary features or the simplifying assumption of symmetry. It is not always necessary to have a full CAD model, depending on the application.

Function is often more important than form. This is reflected in the PLM system, which encourages the provision of detailed design specifications in the early stages of the project. Attempting a slavish reproduction of the overall shape of the original part is not guaranteed to deliver satisfactory performance. A combination of forward and reverse engineering may be more powerful, as this would ensure that only the most essential aspects of the shape would be reproduced, and the function would be emphasised.

Where it is necessary to create a model with accurate dimensions, this would be greatly facilitated if the scanner were to be calibrated prior to use with a certified standard. This would eliminate the need to scale the models using manual measurements, which is a potential source of error. However, even if the model is accurately scaled, it is difficult to establish the permitted tolerance of machining without access to full technical documentation or a large number of example parts. In some cases, dimensions and tolerances are specified in ISO standards, such as conical connectors for anaesthetic and respiratory equipment (ISO 5356-1:2015), and this could potentially eliminate the need to take measurements.

Another difficulty encountered was the scanning of transparent objects. Chalk spray can enable transparent objects to be scanned but CT scanning is often necessary in such cases, as shown here. For the purposes of this paper, a medical grade CT scanner was used due to its ready accessibility, but the resolution of this system was limited. However, in many cases, optical/CT scanning is unnecessary and conventional measurements using Vernier callipers etc are sufficient.

As already noted, attempting to reverse engineer any part, medical or otherwise, presents potential legal issues due to the possibility of intellectual property infringement, with reference to patents or design rights. In some jurisdictions it is possible for legislation to be activated to enable production of proprietary parts by other manufacturers during emergencies but this does not always happen. The situation is often far from clear and in some cases, it is very challenging to find out whether there is a risk of infringement. For example, in the case of the plasticware for testing, the method had been protected by a patent but this had expired by the time of the COVID emergency. Reverse engineering components with expired patents can be valuable, as such products have been thoroughly tested and are highly familiar to users. However, even in the absence of a current patent there may still be some form of intellectual property protection for the components, perhaps in the form of design rights, and without in-depth legal advice it would be difficult to know whether it was safe to proceed. Some of the technical and legal issues could potentially be overcome by a strong partnership with the original manufacturer.

The other major legal issue is compliance with medical device regulations, the landscape for which is complex and changing. Before production of any item for real world use, it is necessary to confirm whether it is or is not a medical device, which regulations apply and what must be done to ensure compliance. To the best of our knowledge, and as confirmed by informal dialogue with the UK regulatory body (MHRA), the syringe pump lockbox does not meet the definition of a “medical device” or “accessory” (62).

MHRA guidance notes that “Modifying existing devices or using them for purposes not intended by the manufacturer (off-label use) has safety implications” and “It is essential that modifications outside of the manufacturer's intended use are only considered as part of a fully documented risk management process within the healthcare organisation's risk management policy and procedures” (63). If due process is observed, it is possible for hospitals to use alternatives for some components. For instance, a viral filter with a cap removed can be used under some circumstances instead of a leak port in a CPAP (Continuous Positive Airway Pressure) NIV system (64, 65).

Medical devices and equipment that are manufactured “in-house” and are to be used clinically, are expected to follow best practice guidance (66). This includes manufacturing as part of an appropriate Quality Management System, such as one that meets the requirements of “*BS EN ISO 13485:2016, Medical devices. Quality management systems. Requirements for regulatory purposes.”* Specialist companies, including consultancies, are very well-equipped to handle the intensive process of medical device development. However, the pandemic has demonstrated that emergency situations may require broader networks of manufacturers and more agile supply chains, which can be rapidly expanded and contracted to meet demand. Agile regulations are required so that adaptive manufacturing can be exploited to the full, and some countries did indeed modify or relax regulations during the COVID-19 emergency (67).

The issues around “In-house” manufacturing are complex when a distributed manufacturing model is considered. In the UK, Medical Devices manufactured by a Health Institution and used by patients within that institution are not considered to have been placed on the market and the Medical Device Regulations do not apply. However, the devices cannot be transferred to another legal entity as this is viewed as placing the devices on the market and CE/UKCA marking is required (68).

In a 2017 draft consultation document, the MHRA provided more detail about what activities are considered “manufacturing,” noting “Items that are used to replace a part or component of a device and that significantly change the performance or safety characteristics or the intended purpose of the device shall be considered to be modification” (69). Institutions that modify a medical device [this includes “use of sample types, accessories or components or combining devices not specified by the (original) manufacturer” (69)] may assume the liability as the manufacturer of the medical device.

In order for replacement parts to be manufactured using a distributed manufacturing model and for this process to be considered as “in-house” manufacturing, it should be made clear that the Health Institution is the legal entity responsible for such manufacturing even when the part is created by a university or commercial partner.

Members of the Physical Healthcare Sciences already produce numerous custom or patient specific devices, including items for surgery, improving patients experience and lifestyle and in training of clinical staff. As clinical services wish to adopt personalised healthcare within the patient pathway, there will be increased demand to design and manufacture a wider range of in-house devices and solutions, using methods such as those presented in this work, to develop solutions and associated design and testing documentation to comply with incoming medical device regulations. In-house production of rapidly prototyped components would not only support the precision medicine agenda, and ensure the supply of essential consumables in a time of emergency, but would help to enable older apparatus to be kept in operation after the Original Equipment Manufacturer has ceased support. Production runs would be small but rapid turnarounds would be needed. By using a PLM system such as that presented here, which allows the workflow to be adapted to the project, operations could be managed effectively and well-documented. As has been shown, adaptive manufacturing has remarkable potential for supporting an emergency response but also for routine production of essential items, in healthcare and other domains.

## Data Availability Statement

The original contributions presented in the study are included in the article/Supplementary Material, further inquiries can be directed to the corresponding authors.

## Author Contributions

MB and AV: optical scanning. ASt and AV: manual measurement of parts. AT: CT scanning. KD, SF, SI, GM, FQ, NP, and AV: design of workflows and processes for adaptive manufacturing. KD, GM, NP, ASh, and AV: implementation of system in aras. PA, SB, SC, SG, MM, AM, GP, and MP: syringe pump lock-box design & production. MB (scan processing), GC (manifold), AJS (plasticware), and ASt (leak port design): scan processing & CAD of reverse-engineered parts. CD, SI, SK, and SR: hardware specification and assessment. GC, JM, and ASt: 3D printing. AD, KD, SF, SG, SI, FQ (Good Document Practice), ASh, and AV: identifying methodology (other than that covered by other headings). AD: regulatory expertise. KD, SG, JM, and AV: supervision. KD: overall project leadership & coordination. GC, KD, JM, NP, AJS, ASt, and AV: writing (original draft & figures). KD and JM: writing (editing). All authors writing (review), contributed to the article, and approved the submitted version.

## Conflict of Interest

Some of the authors are affiliated with companies that supply products or services that could be used for the applications described in this paper or related work. GM is Vice President Operations of AESSiS, an Aras Certified Systems Integration Partner for 11 years. The remaining authors declare that the research was conducted in the absence of any commercial or financial relationships that could be construed as a potential conflict of interest.

## Publisher's Note

All claims expressed in this article are solely those of the authors and do not necessarily represent those of their affiliated organizations, or those of the publisher, the editors and the reviewers. Any product that may be evaluated in this article, or claim that may be made by its manufacturer, is not guaranteed or endorsed by the publisher.
