# Supplementary material for: Adaptive Manufacturing for Healthcare During the COVID-19 Emergency and Beyond
Source: Front Med Technol. 2021 Aug 2;3:702526. doi: 10.3389/fmedt.2021.702526 (PMC8757720; doi:10.3389/fmedt.2021.702526)
Supplement: Supplementary file 1 [file Data_Sheet_1.PDF]

Supplementary Material

1 Supplementary Information 1: Instructions for using the PLM system in Aras Innovator (co-ordinator's manual)

1.1 General information

This document outlines the steps that must be followed by a coordinator for every job. No Admin privileges are required unless explicitly specified.

Connect to Aras via a web browser via the designated URL.

Enter your username and password and click login.

The first time you login, the page might take some time to load.

When the page successfully loads, a page called MyInBasket should load. An example of the landing page would be:

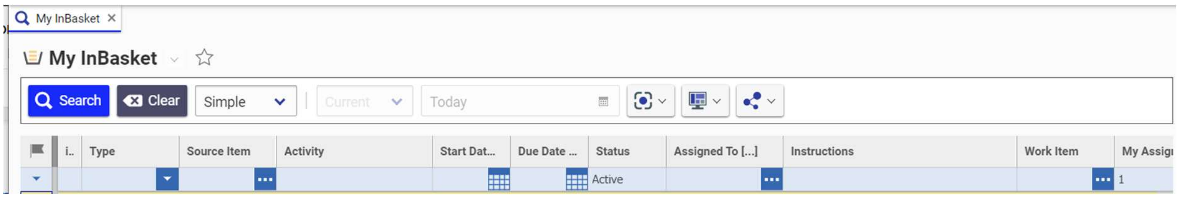

An authentication bug exists where the loading screen is blank and will not load. To fix this issue, truncate the URL of your browser and hit enter.

1.2 Searching in Aras:

Searching a list is very common in Aras. Search criteria can be added at a search field to filter the list. For example, when searching for jobs, a large list of jobs appears. Use the first row (blue) to enter filters. (image truncated):

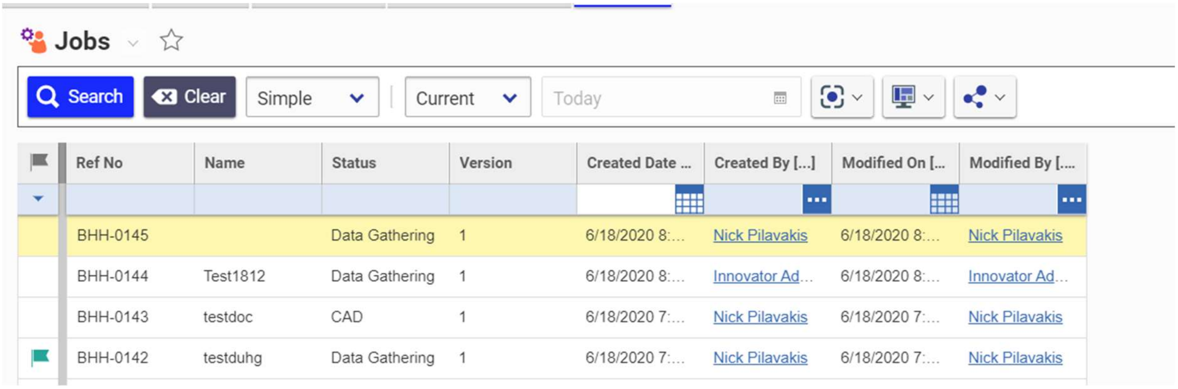

To execute a search, press ENTER 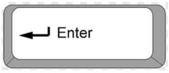

or click Search 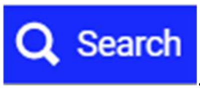

.

For simplicity, only the most common rules will be outlined:

1. Search is case insensitive. For example to find the job with ref number BHH-0143:

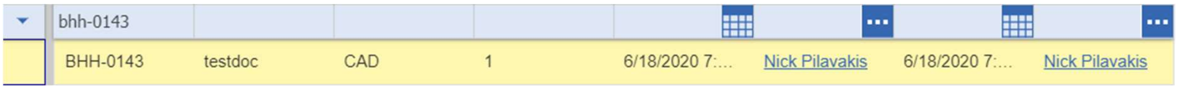

2. Text search utilises pattern matching. This means that an exact (but case insensitive) match must be found for a row to be returned. Multiple special characters exist in pattern matching. The most important one is the asterisk (*) which denotes any pattern. For example searching for "a*" would return all rows for which the specified column starts with the letter a. On the contrary, "*a" would return all rows for which the specified column ends with the letter a. To return the job with ref number BHH-0143 we can search using *143

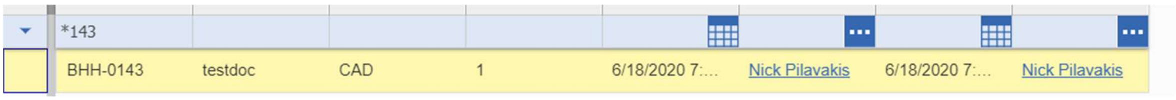

3. Clicking on a column sorts the results in ascending order, clicking it again sorts the results in descending order.

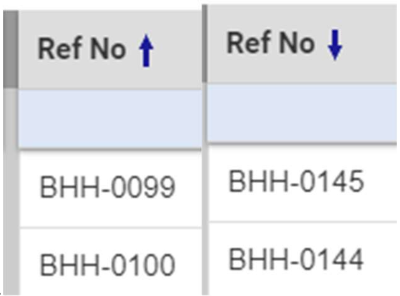

4. If filters are added to multiple columns, all filters must be satisfied for the row to be returned.

1.3 Creating a job:

1) Display the grid by clicking on the red grid icon on the top left corner: 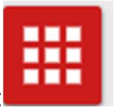
2) Click on jobs 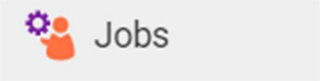

3) Click create new job 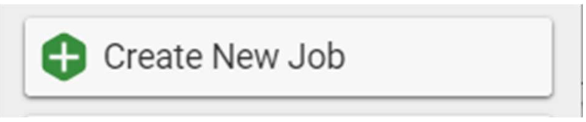

4) If applicable, assign a name to the job by typing it to the corresponding text box:

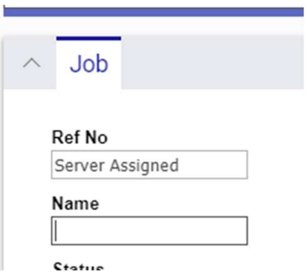

5) Click Done. 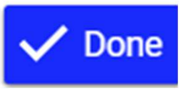

1.4 Finding a job:

1. Display the grid by clicking on the red grid icon on the top left corner: 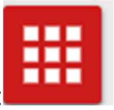
2. Click on jobs 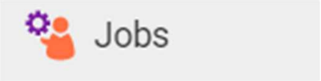

3. Click search jobs 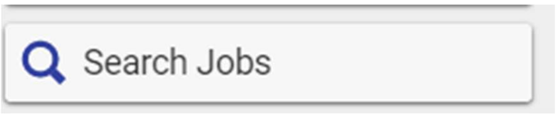

4. Double click on the row representing the job of interest. The job will then open in a new Aras tab.

1.5 Assigning roles to a job:

1. Find the job (see relevant section for help)
2. Click edit (top left) 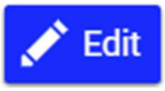

3. Click the Assignments tab 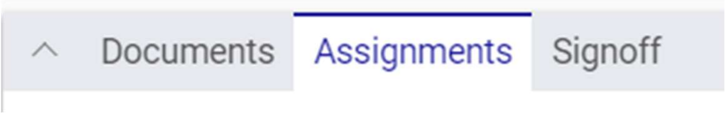

4. If the person to be assigned to the role is the person shown on the “default user” column, nothing needs to be done.
5. If the default is not the right person for this job, double click on the “user” column of the row of interest. A white text box will appear

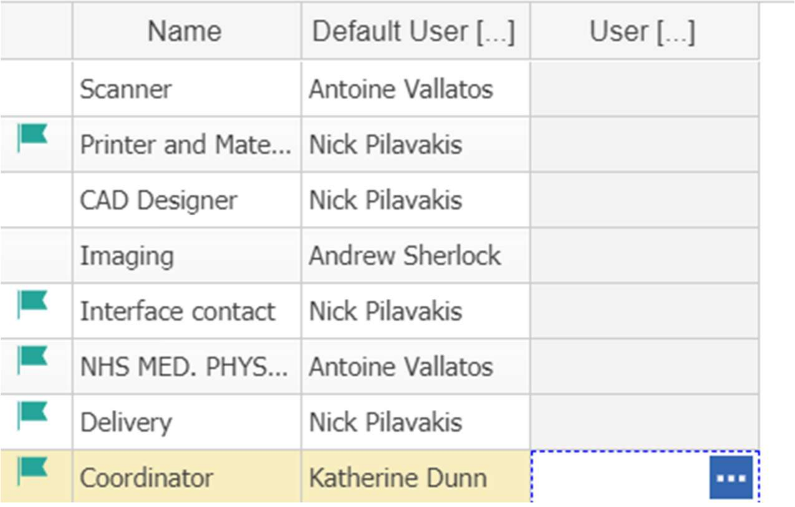

	Name	Default User [...]	User [...]
	Scanner	Antoine Vallatos	
	Printer and Mate...	Nick Pilavakis	
	CAD Designer	Nick Pilavakis	
	Imaging	Andrew Sherlock	
	Interface contact	Nick Pilavakis	
	NHS MED. PHYS...	Antoine Vallatos	
	Delivery	Nick Pilavakis	
	Coordinator	Katherine Dunn	

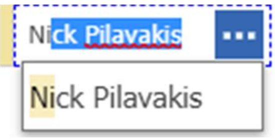

6. Start typing the name of the user. Suggestions will appear.
7. If the correct person is auto completed, press enter to select that person. Alternatively, the name of the required person can be selected by clicking the name from the drop down list.
8. If the required person does not show up, click on the three dots 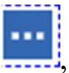, a search menu will appear. Refer to section "Searching in Aras" for details. Double click on the required person to select
9. Repeat the above steps for as many roles required.
10. Click done 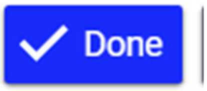

1.6 Progressing a workflow:

Progressing a workflow can happen through two different channels - the signoff tab or the inbasket. Both provide exactly the same functionality, the only difference is the way the data is presented.

To progress a workflow through the signoff tab, find the job and click on the signoff tab.

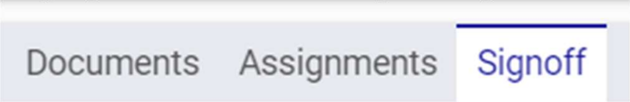

Here you can see the progress of the most recent workflow. If the latest activity in the current workflow is assigned to you, you get the option to vote (VOTE NOW link) in order to progress the

workflow to the next activity.

Activity	State	Assigned To	Completed By	How Voted
Submit	Active	Nick Pilavakis		VOTE NOW

To progress a workflow through the inbasket:

1) Display the grid by clicking on the red grid icon on the top left corner: 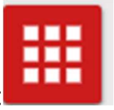

2) Click "My InBasket" 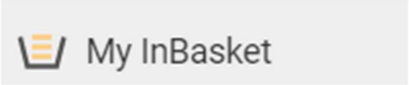

3) Click "Search My InBasket" 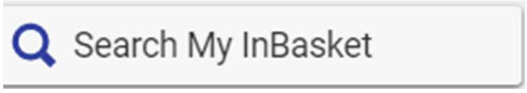

Here, you can see all the activities that you can currently vote on. Double clicking on a row allows you to vote for the corresponding job.

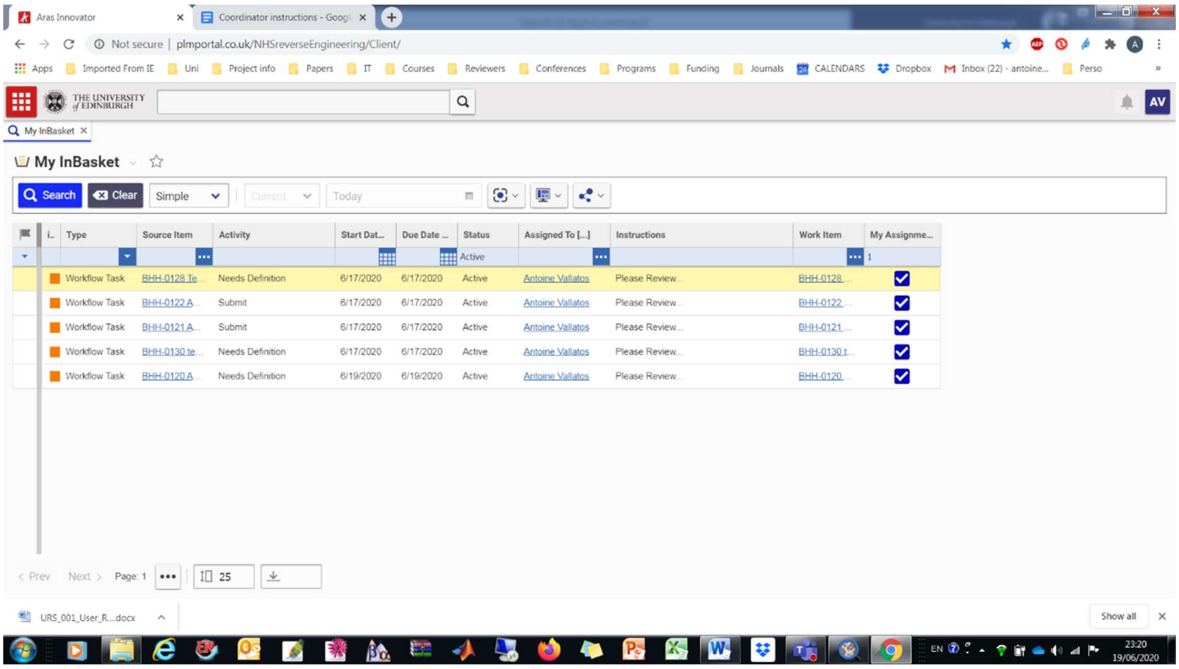

1.7 Voting:

To vote for a job, use either of the two methods outlined in the previous section.

When voting, the following window appears:

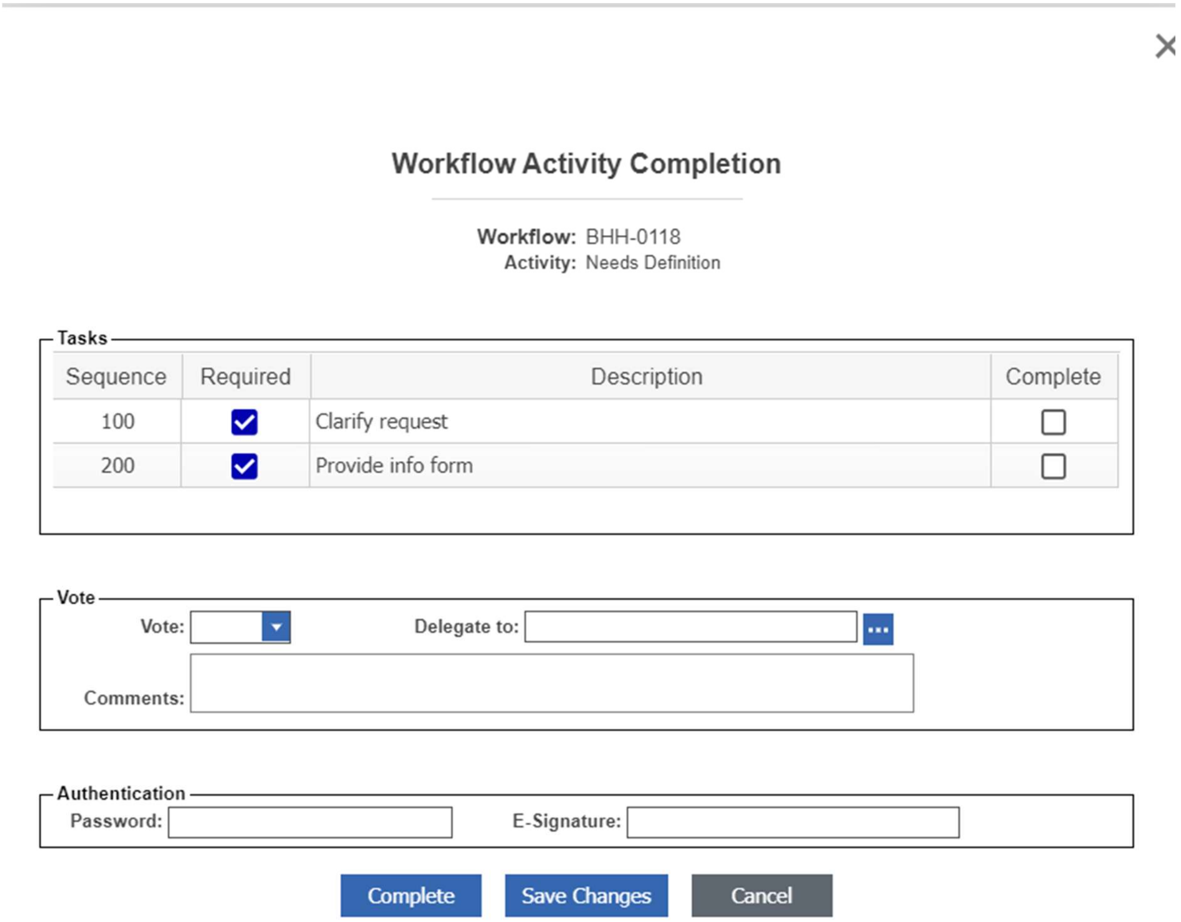

Some activities, such as this one contain multiple tasks. Completion of some of these tasks is mandatory in order to proceed and are marked with a tick on the required column. Mark the Completed tasks by clicking the box on the “Complete” column.

Click on the box next to vote to display the drop down list with the available options. Most activities only allow for one option. Some activities allow for two options. The selected option dictated the path that the workflow will dictate. After selecting a vote, optionally add any comments. To save your progress and come back later click on “Save changes”. To move the workflow, click on Complete.

1.8 Resetting the workflow: (Admin only)

If a mistake was made in the current workflow, there is an option to restart it. This will erase any current progress in the current workflow, maintaining the current lifecycle state. This means that the workflow will still need to be progressed to the desired activity.

To reset the workflow:

Find the required job (See, Finding a job)

1. Click the three dots. A drop down menu will appear.

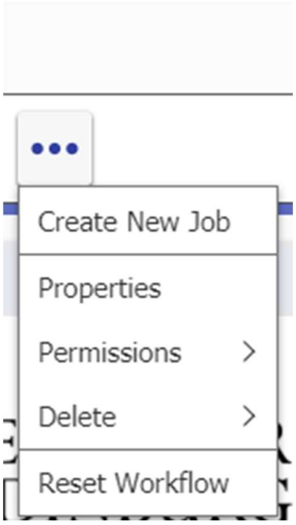

Click Reset workflow

For the changes to show at the signoff tab, the job must be closed and reopened, to close the job

Click the little x at the right end of the tab. 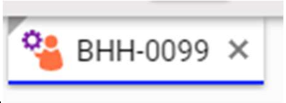

Open the job again

1.9 Handling documents:

1) Find the job of interest as described in the "Finding a job" section
2) Click on the documents tab 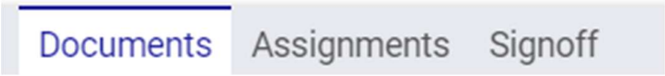

3) After the first activity is completed, 8 document templates will appear. If you wish to add a different document, refer to the next section.
4) Double click on the row of the document of interest to open it.
5) To download a document, click on the download file button 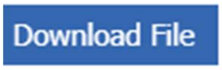

6) To upload a document, click on the edit button 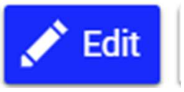

and then click on upload file 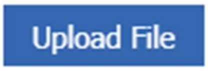

Select the file you wish to upload.
7) Notice that the upload/download activity is registered at the file history tab

1.10 Add new document to job:

1) Find the job
2) Click on the edit button: 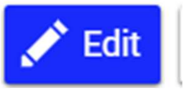

3) Click on the documents tab 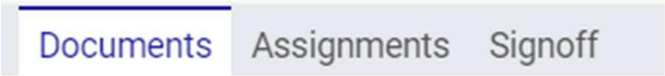

4) Click on the create Item button. (White cross green background) 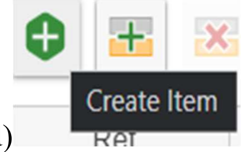

5) A new row will be created. Click done 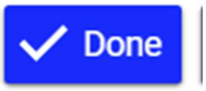

6) Double click on the newly created row. The document will open on a new tab.
7) Click on the edit button: 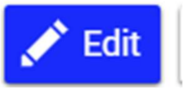

8) Add a document name,optionally add a classification
9) Click on save 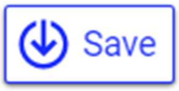

10) Click on upload file and upload the required file. 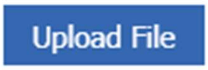

2 Supplementary Information 2: Forms and Documents created for PLM system

The following forms are provided for illustrative purposes. Forms similar to these were used to test the Aras system. Further project-specific development would be required.

The user requirements form is intended to support collection of information from the clinical engineers/medical physicists who are requesting the part.

The purpose of the fill-in form is to document that an event has occurred e.g. production of the technical specifications.

The standard operating procedure is a methodology document that would be completed to describe exactly how an individual step (e.g. optical scanning or 3D printing etc) would be performed.

User requirements form

Date:

NHS Contacts (please include phone number when available):
NHS Site (Part Location):

Part name:
Part provider:
Reason for replacement (single use part/broken/other):
Number of parts required:
Emergency level:
Comments:

Usage

- In situ description with pictures (as many as possible): YES/NO
- Interfaces with other components: YES/NO
- Function (cover/mechanical function): YES/NO
- In contact with chemicals (define chemicals): YES/NO
- In contact with inhaled/injected gazes/drugs: YES/NO
- Mounting/Unmounting frequency:

Comments:

Design & Material

Material:
CAD availability: YES/NO
Optical 3D scan availability: YES/NO
Possibility to arrange onsite 3D scan (part can be sterilised/made available): YES/NO
Possibility to take the part out of site: YES/NO
Comments :

Dimensions/Constrains:

- Critical dimensions:
- Sealed surfaces:
- Applied forces:

Comments:

Sterilisation needs:

Fill in form

Type of form:

☐ Job specifications

☐ Computer Aided Design

☐ Printing protocol

☐ In situ validation

Description:

Information to be recorded (i.e. Final Job specifications, next CAD or printing protocol version, In situ validation outcomes).

Approvals:

Name	Signature Reason	Function/ Department	Date	Signature

Revision history:

Revision Number	Date	Name of Author	Description of Change

Job Production Workflow

Standard Operating Procedure

Part name:

Project ID:

Approvals:

Name	Signature Reason	Function/ Department	Date	Signature
	Author			
	Reviewer			
	Approver			
	Approver			

Revision history:

Revision Number	Date	Name of Author	Description of Change

Previous version:

Reason for change:

Regulatory basis and reference documents

List of background references

Purpose

Why is this procedure to be performed?

Scope

Description of what is covered by this SOP

Procedure

Step-by-step protocol, specifying exactly what is to be done such that the procedure will be performed in exactly the same way each time

Responsibilities

Define roles within this operation

Additional Instructions

Non-workflow related tasks

Abbreviations

If applicable

Annexes/ Related Forms

- USR form
- FRM versions for CAD, printing and in situ validation
